# PNPO‐Mediated Oxidation of DVL3 Promotes Multiple Myeloma Malignancy and Osteoclastogenesis by Activating the Wnt/β‐Catenin Pathway

**DOI:** 10.1002/advs.202407681

**Published:** 2024-12-10

**Authors:** Zhendong Deng, Shanliang Sun, Nian Zhou, Yumeng Peng, Long Cheng, Xichao Yu, Yuxia Yuan, Mengjie Guo, Min Xu, Yuexin Cheng, Fan Zhou, Nianguang Li, Ye Yang, Chunyan Gu

**Affiliations:** ^1^ Nanjing Hospital of Chinese Medicine affiliated to Nanjing University of Chinese Medicine Nanjing 210022 China; ^2^ School of Medicine Nanjing University of Chinese Medicine Nanjing 210023 China; ^3^ National and Local Collaborative Engineering Center of Chinese Medicinal Resources Industrialization and Formulae Innovative Medicine Nanjing University of Chinese Medicine Nanjing 210023 China; ^4^ Department of Hematology and Oncology Jing'an District Zhabei Central Hospital Shanghai 200070 China; ^5^ Yangtze River Delta County Hematology Union Shanghai 200070 China; ^6^ Department of Hematology Zhangjiagang First People's Hospital Zhangjiagang 215600 China; ^7^ Department of Hematology Yancheng Clinical College of Xuzhou Medical University Yancheng No.1 People's Hospital Yancheng 224006 China

**Keywords:** dishevelled 3, eltrombopag, multiple myeloma, pyridoxine‐5′‐phosphate oxidase, wnt/β‐catenin pathway

## Abstract

Multiple myeloma (MM) is a cancer of plasma cells caused by abnormal gene expression and interactions within the bone marrow (BM) niche. The BM environment significantly influences the progression of MM. Celastrol, a natural compound derived from traditional Chinese medicine, exhibits significant anticancer effects. This study aimed to identify specific targets of celastrol and develop more effective and less toxic treatment options for MM. Celastrol is used as a probe to determine its specific target, pyridoxine‐5′‐phosphate oxidase (PNPO). Increased levels of PNPO are associated with poor outcomes in MM patients, and PNPO promotes MM cell proliferation and induces osteoclast differentiation through exosomes. Mechanistically, PNPO oxidizes disheveled 3^M282^ (DVL3), leading to abnormal activation of the Wnt/β‐catenin pathway. Based on the critical sites of PNPO^R95/K117^, Eltrombopag is identified as a potential therapeutic candidate for MM. In addition, the experiments showed its efficacy in mouse models. Eltrombopag inhibited the growth of MM cells and reduced bone lesions by disrupting the interaction between PNPO and DVL3, as supported by preliminary clinical trials. The study highlights the importance of PNPO as a high‐risk gene in the development of MM and suggests that Eltrombopag may be a promising treatment option.

## Graphical abstract

Pyridoxine‐5'‐phosphate oxidase (PNPO), a target of celastrol, promotes cell proliferation and osteoclast differentiation via exosomes in multiple myeloma (MM). PNPO oxidizes dishevelled 3^M282^ (DVL3), leading to aberrant activation of the Wnt/β‐catenin pathway and upregulation of MYC. Eltrombopag disrupts the interaction between PNPO and DVL3 to inhibit MM cell growth and reduce bone lesions, as supported by preliminary clinical trials.

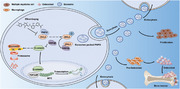

## 1 Introduction

Multiple myeloma (MM), the second most common hematological malignancy originating from terminally differentiated B lymphocytes in the bone marrow, is characterized by symptoms such as bone damage and anemia.^[^ ^1^, ^2^ ^]^ The development of MM is heavily influenced by communication with the bone marrow (BM) environment,^[^ ^3^ ^]^ which can worsen patient prognosis and lead to drug resistance. MM cells have high levels of integrin‐α4β1^[^ ^4^ ^]^ and CD44,^[^ ^5^ ^]^ which allow them to enter the BM and stimulate the secretion of IL6,^[^ ^6^ ^]^ promoting their proliferation and inducing monocyte osteoclast differentiation, ultimately leading to immune escape.^[^ ^7^ ^]^ Despite advancements in treatment, the median survival of MM patients remains less than eight years, potentially due to the limitations of current therapies that only target MM cells. Therefore, it is crucial to find new strategies that can target both MM cells and the BM simultaneously.

Traditional Chinese medicine (TCM) has been used for thousands of years and is effective in treating cancer. For example, compounds such as nitidine chloride^[^ ^8^ ^]^ and quercetin,^[^ ^9^ ^]^ derived from *Zanthoxylum nitidum* and *Ambrette*, have been proven to directly inhibit MM cell proliferation or improve the BM environment to alleviate patient suffering. Our current focus is on celastrol, a compound extracted from *Tripterygium Wilfordii* that has shown promising anticancer effects in various cancers, including MM, by inhibiting NF‐κb and STAT3 activation^[^ ^10^ ^]^ or proteasome activity.^[^ ^11^ ^]^ Additionally, celastrol has been shown to improve the BM microenvironment,^[^ ^12^ ^]^ making it a precious compound for treating MM. Importantly, however, celastrol also has potential side effects and toxicities, such as cardiac,^[^ ^13^ ^]^ hepatic,^[^ ^14^ ^]^ hematopoietic,^[^ ^15^ ^]^ reproductive,^[^ ^16^ ^]^ and renal^[^ ^17^ ^]^ toxicity, which limit its clinical application. To address this challenge, we conducted rigorous experiments to identify the specific targets of celastrol in MM, targeting both MM cells and the BM environment. Through fluorophore labeling and microarray analysis of patient cohorts, we used celastrol as a probe labeled with FITC as a probe to screen for potential targets. Ultimately, we identified pyridoxamine‐5′‐phosphate oxidase (PNPO) as a potential target for both MM cell proliferation and bone damage, as observed in microarray cohorts of MM patients.

PNPO (EC 1.4.3.5) is an oxidase that plays a crucial role in the metabolism of vitamin B6 and acts as a cofactor for over 140 enzymes,^[^ ^18^, ^19^ ^]^ which is widely distributed in various tissues.^[^ ^20^ ^]^ However, excessive expression of PNPO is associated with tumor progression,^[^ ^21^ ^]^ leading to increased proliferation and metastasis of cancer cells, such as in breast cancer^[^ ^22^ ^]^ and ovarian cancer.^[^ ^23^ ^]^ Despite this, more research is needed on the role of PNPO in MM. Furthermore, post‐translational modifications, essential steps in protein synthesis, have been found to play significant roles in the progression of MM.^[^ ^24^, ^25^, ^26^ ^]^ Oxidation, an essential function of PNPO, is a common post‐translational modification that can significantly impact cell fate. Moreover, inhibitors of other oxidases, such as cholesterol oxidase (CO) and lysyl oxidase, have been shown to inhibit tumor growth and metastasis effectively.^[^ ^27^ ^]^ However, there is limited research on the oxidation function of PNPO in cancers, particularly in MM. Our ultimate goal is to utilize this target structure to develop a novel therapeutic drug with minimal side effects.

In this study, we identified PNPO as a target of celastrol and validated its role in MM cell proliferation and osteoclast differentiation through oxidation. Furthermore, we examined the potential therapeutic effects of Eltrombopag as a PNPO inhibitor.

## 2 Results

### 2.1 PNPO is a High‐Risk Indicator for Poor Prognosis and Promotes Cell Proliferation in MM

**Figure 1:**
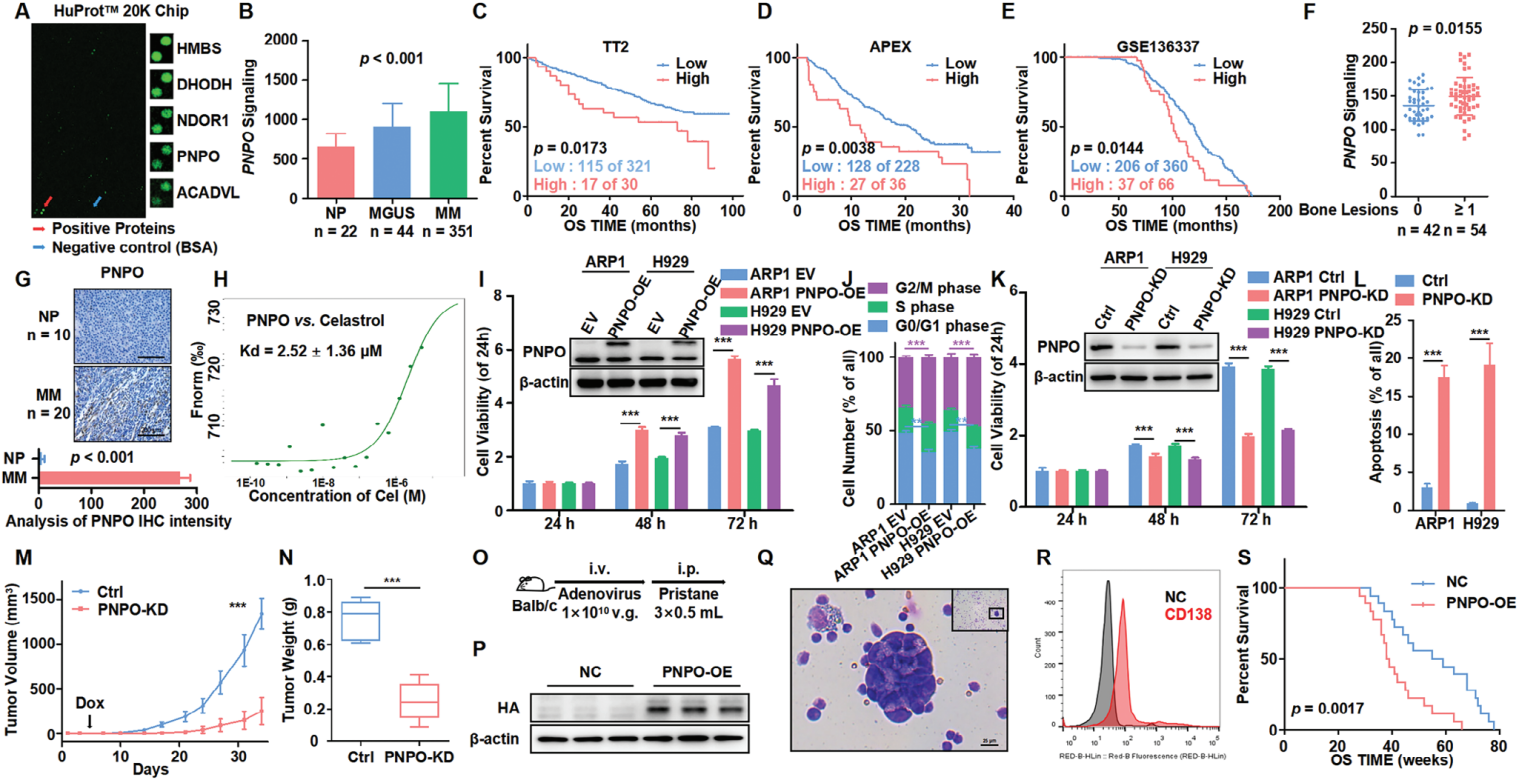
PNPO is a high‐risk indicator for poor prognosis and promotes cell proliferation in MM. A) The HuProt 20K Proteome Microarray Chip revealed the top 5 protein targets binding to Celastrol. The red arrow indicates positive protein binding to Celastrol, and the blue arrow indicates the negative control. B) PNPO mRNA levels were significantly increased in MM patients from the GSE5900 dataset. The level of PNPO is shown on the *y*‐axis, while the patients with NP (*n* = 22), MGUS (*n* = 44), and MM (*n* = 351) are displayed on the *x*‐axis. C–E) Increased PNPO mRNA expression was positively associated with poor overall survival (OS) in MM patients from TT2 C), APEX D), and GSE136337 E) datasets. F) PNPO mRNA levels were high in patients with bone lesions from the GSE6401 dataset. G) Representative IHC staining of primary MM (*n* = 20) and NP (*n* = 10) samples, along with statistical analysis of PNPO IHC staining intensity between the NP and MM groups. H) MST analysis of the interaction between PNPO protein and celastrol. I) WB examined PNPO expression in EV and PNPO‐OE ARP1 and H929 cells, and CCK8 assay showed greater proliferation capacity of PNPO‐OE MM cells compared to EV MM cells (*n* = 5). J) Cell cycle analysis of EV and PNPO‐OE cells (*n* = 3). K) WB examined PNPO expression in Ctrl and PNPO‐KD MM cells, and CCK8 assay showed a weaker proliferation capacity of PNPO‐KD MM cells compared to Ctrl cells (*n* = 5). L) Cell apoptosis analysis for Ctrl and PNPO‐KD cells (*n* = 3). M) Time course of tumor growth in H929 Ctrl and PNPO‐KD xenograft NOD‐SCID mice (*n* = 5). N) Tumor weights of H929 Ctrl and PNPO‐KD xenografts taken from NOD‐SCID mice (*n* = 5). O) Schematic diagram of establishing a pristane‐inducible mouse model. P) WB detected HA‐tag expression in spleen tissues. Q) Giemsa staining demonstrated a plasmacytoid appearance of ascite cells. R) Flow cytometry revealed that ascites cells universally expressed CD138, a marker of plasma cells. S) PNPO overexpression decreased the survival of pristane‐inducible model mice (*n* = 18). Data are represented as mean ± SD. ***p < 0.001. Bars represent 200 (G) and 25 µm (Q).

We utilized a fluorescent probe, FITC, to label celastrol and conducted a HuPro20K Proteome Microarray Chip to identify potential therapeutic targets for MM. Our analysis revealed 45 proteins with a significant binding affinity for celastrol (**Figure** **1**A). Further analysis of GEP data from MM patient cohorts identified PNPO as the top candidate associated with MM progression. PNPO expression was significantly elevated in patients with monoclonal gammopathy of undetermined significance (MGUS, *n* = 44) and MM (*n* = 351) compared to normal plasma (NP, *n* = 22) (*p* < 0.001) (Figure 1B). Furthermore, high PNPO expression was associated with inferior outcomes in TT2 (*p* = 0.0173) (Figure 1C), APEX (*p* = 0.0038) (Figure 1D), and GSE136337 (*p* = 0.0144) (Figure 1E) cohorts. Interestingly, we also observed high PNPO expression in patients with bone lesions (*p* = 0.0155) (Figure 1F). IHC assay further confirmed strong PNPO expression in primary MM tissues (Figure 1G) and a positive correlation with Ki67, a key marker of cell proliferation (*p* < 0.001, r^2^ = 0.819) (Figure S1A, Supporting Information). Additionally, MST analysis confirmed a direct interaction between celastrol and PNPO (Figure 1H).

To further investigate the role of PNPO in MM cell lines, we utilized a lentiviral system to establish stable overexpression of PNPO in ARP1 and H929 cell lines. WB analysis confirmed the successful overexpression of PNPO, and CCK8 assay showed that increased PNPO expression significantly promoted MM cell growth (*p* < 0.001) (Figure 1I). The results from the cell cycle analysis revealed that a greater proportion of PNPO‐OE cells was in the G2/M phase (*p* < 0.001) (Figure 1J; Figures S1B,C, Supporting Information). Conversely, PNPO‐KD cells generated via the use of PNPO‐inducible‐targeting shRNAs presented a significantly lower cell growth rate compared to control (Ctrl) cells (*p* < 0.001) (Figure 1K). Additionally, the knockdown of PNPO led to increased cell apoptosis (*p* < 0.001) (Figure 1L; Figures S1D,E, Supporting Information).

To validate these findings in vivo, we injected H929 PNPO‐KD cells into NOD‐SCID mice (Figure S1F, Supporting Information). After four weeks, we observed that the MM xenograft tumors formed by PNPO‐KD cells were visibly smaller than those formed by PNPO‐Ctrl cells (*n* = 5, *p* < 0.001) (Figures 1M,N; Figures S1G,H, Supporting Information), whereas the use of Dox had no significant effect on mouse weight (Figure S1I, Supporting Information). WB analysis confirmed the downregulation of PNPO in the tumor tissues (Figure S1J, Supporting Information). We also investigated the impact of PNPO on disease progression using a pristane‐induced model, a well‐established model for spontaneous plasma cell tumors (Figure 1O). WB analysis confirmed the overexpression of PNPO tagged with HA (Figure 1P). At 18 weeks, we observed the presence of ascites, which gradually increased and caused abdominal distention. Giemsa staining revealed a plasmacytoid appearance of the ascite cells (Figure 1Q). Flow cytometry and immunofluorescence (IF) analyses showed that the ascites cells expressed CD138, a marker of plasma cells (Figure 1R; Figures S1K,L, Supporting Information). Additionally, serum protein electrophoresis (SPEP) revealed abnormal results in most mice (Figure S1M, Supporting Information). Ultimately, the overexpression of PNPO significantly decreased the survival of the mice (*n* = 18, *p* = 0.0017) (Figure 1S). These in vivo results further support the role of PNPO as a high‐risk factor that promotes cell proliferation in MM.

### 2.2 PNPO Promotes Osteoclast Differentiation via Exosomes

**Figure 2:**
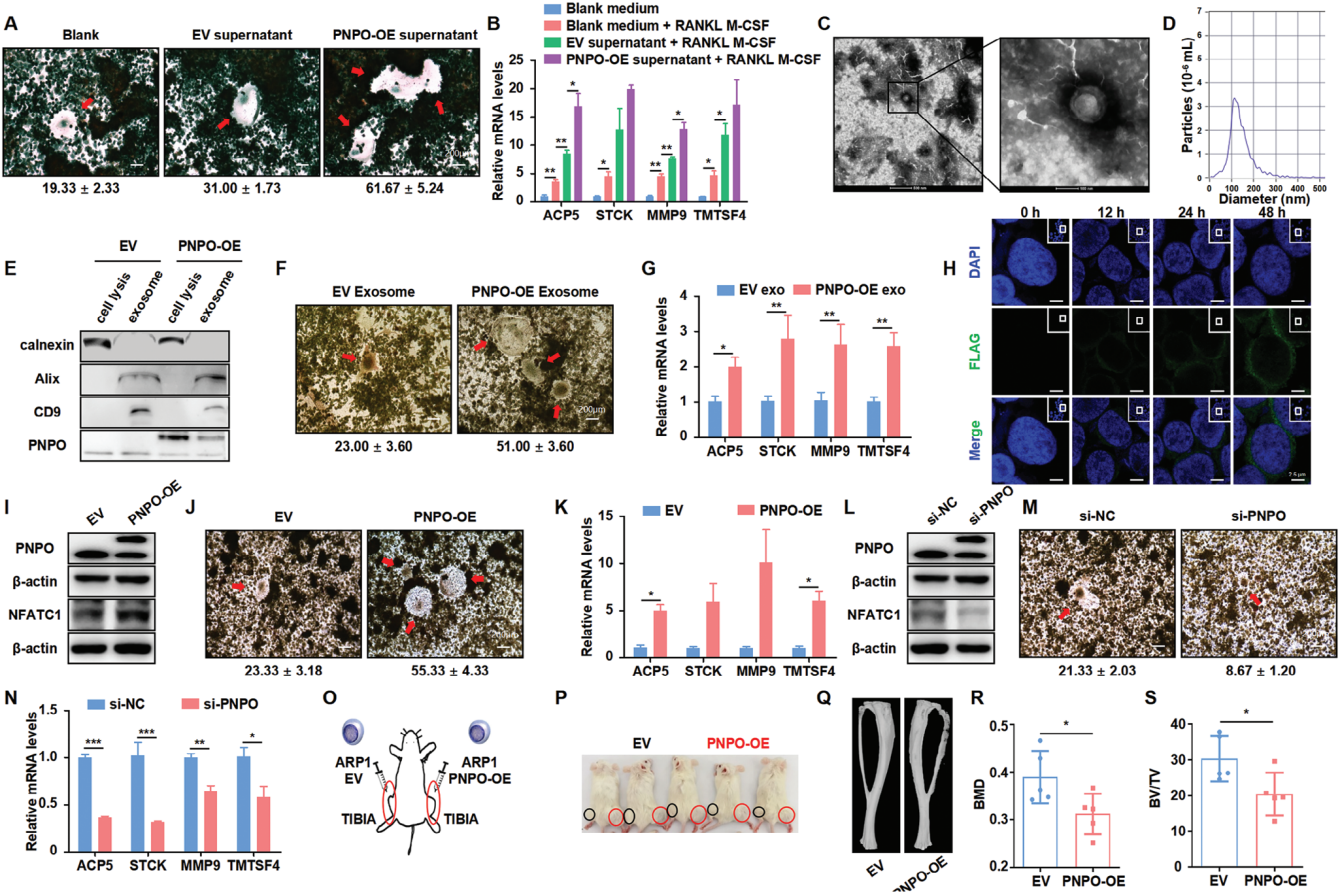
PNPO promotes osteoclast differentiation via exosomes. A) TRAP activity assay showed that the supernatant from PNPO‐OE MM cells promoted the differentiation of RAW264.7 cells into osteoclasts (*n* = 3). B) The expression of osteoclast‐related mRNAs was detected in RAW264.7 cells treated with or without supernatant from PNPO‐OE MM cells (*n* = 3). C) The biological characteristics of exosomes were detected via transmission electron microscopy. D) Exosomes were analyzed using NTA. E) Calnexin, Alix, CD9, and PNPO expressions were examined via WB analysis. F) TRAP activity results revealed that exosomes from PNPO‐OE MM cells promoted the differentiation of RAW264.7 cells into osteoclasts (*n* = 3). G) The expression of osteoclast‐related mRNAs was detected in RAW264.7 cells treated with exosomes from EV and PNPO‐OE MM cells (*n* = 3). H) Confocal microscopy revealed a time‐dependent increase of PNPO expression in ARP1 WT cells. I) WB analysis showed the expression of PNPO and NFATC1 in RAW264.7 EV and PNPO‐OE cells. J) TRAP activity results revealed that elevated PNPO promoted the differentiation of RAW264.7 cells into osteoclasts (*n* = 3). K) The expression of osteoclast‐related mRNAs was detected in RAW264.7 EV and PNPO‐OE cells (*n* = 3). L) WB analysis showed the expression of PNPO and NFATC1 in si‐NC and si‐PNPO RAW264.7 cells. M) TRAP activity results revealed that silencing PNPO inhibited the differentiation of RAW264.7 cells into osteoclasts (*n* = 3). N) The expression of osteoclast‐related mRNAs was detected in RAW264.7 si‐NC and si‐PNPO cells (*n* = 3). O) A schematic diagram of the NOD‐SCID TIBIA model. P) Photographic images of NOD‐SCID TIBIA mice were taken at the end of the experiment. Q–S) Representative microCT images Q), BMD R), and BV/TV S) of NOD‐SCID TIBIA mouse bones in the EV and PNPO‐OE groups (*n* = 5). Data are represented as mean ± SD. *p < 0.05, **p < 0.01, ***p < 0.001. Bars represent 2.5 µm (H), 200 µm (A,F,J), and (M), and 500 or 100 nm (C).

After GEP analysis, a correlation was found between bone damage and PNPO expression. The malignant proliferation and differentiation of osteoclasts (OCs) in the BM microenvironment led to exacerbations and shortened survival in MM patients. Furthermore, we utilized pre‐osteoclast RAW264.7 cells to investigate the link between PNPO and osteoclast differentiation. TRAP staining showed that the supernatant from ARP1 PNPO‐OE cells significantly promoted the differentiation of RAW264.7 cells into OCs, compared to ARP1 EV cells (*p* = 0.0159) (**Figure** **2**A), and the expressions of OC‐related genes were upregulated (Figure 2B).

Exosomes (Exos) are essential components of the BM microenvironment and have a strong ability to induce osteolysis. We isolated Exos from ARP1 EV and PNPO‐OE cells via ultracentrifugation. Transmission electron microscopy demonstrated a cup‐plate‐like structure of the Exos (Figure 2C), and nanoparticle tracking analysis confirmed that their diameter was ≈100 nm (Figure 2D). WB analysis showed the presence of PNPO and exosomal markers Alix and CD9 in Exos. However, the endoplasmic reticulum (ER) marker calnexin was not detected in Exos (Figure 2E). TRAP staining assay revealed that Exos derived from ARP1 PNPO‐OE cells significantly increased osteoclast differentiation compared to Exos derived from ARP1 EV cells (*p* = 0.0054) (Figure 2F). The mRNA levels of the osteoclast differentiation marker were also increased in Exos derived from ARP1 PNPO‐OE cells compared to Exos derived from ARP1 EV cells (*p* < 0.05) (Figure 2G). To investigate potential cell‐to‐cell communication, we performed a coculture experiment using hanging cell culture inserts, as previously described.^[^ ^28^ ^]^ PNPO migrated from ARP1 PNPO‐OE cells into ARP1 WT cells (Figure 2H).

To further establish the connection between PNPO and osteoclast differentiation, we overexpressed PNPO in RAW264.7 cells (Figure 2I). Our results showed that increased levels of PNPO promoted osteoclast differentiation (*p* = 0.0036) (Figure 2J) and also led to an increase in the relative mRNA levels of osteoclast differentiation marker (Figure 2K). Conversely, silencing PNPO expression (Figure 2L) significantly reduced osteoclast differentiation (*p* = 0.0058) (Figure 2M) and decreased the relative mRNA levels of the osteoclast differentiation marker (Figure 2N). To further investigate the role of PNPO in osteoclast differentiation, we injected ARP1 EV and PNPO‐OE cells into the BM cavity of tibias in NOD‐SCID mice, respectively (Figure 2O). The mice exhibited significant lameness and more pronounced swelling on the right side compared to the left side (Figure 2P). We also used µCT to analyze the bone density and volume of the tibia. The microCT images showed that bone damage was significantly more severe in the PNPO‐OE group compared to the EV group (Figure 2Q). Additionally, the bone mineral density (BMD) and bone volume (BV/TV) were remarkably decreased in the PNPO‐OE group (*n* = 5, *p* < 0.05) (Figures 2R,S). These results suggest that PNPO plays a crucial role in osteoclast differentiation through the delivery of exosomes.

### 2.3 PNPO Affects Cell Proliferation and Osteoclast Differentiation by Activating the Wnt/β‐Catenin Pathway in MM

**Figure 3:**
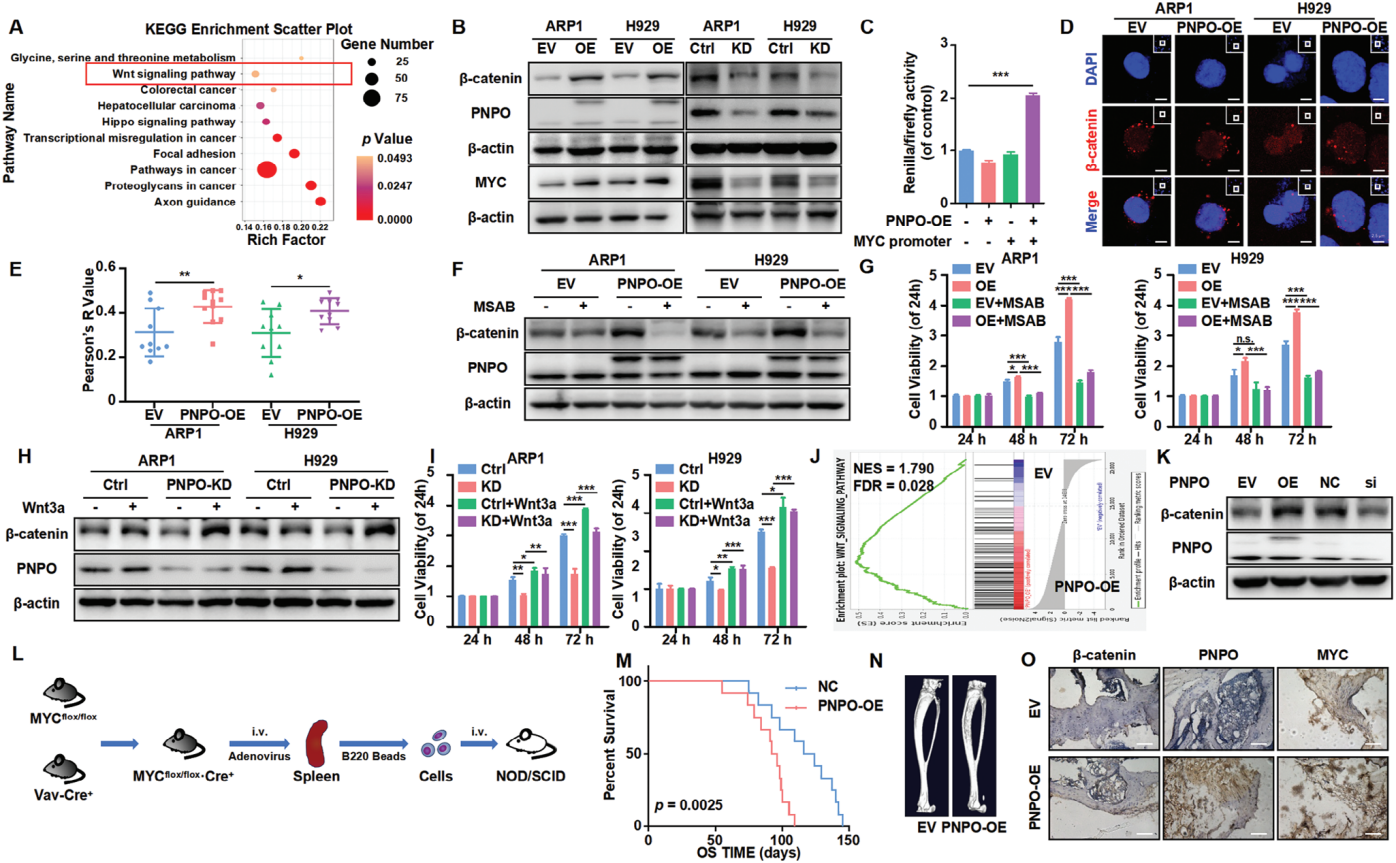
PNPO affects cell proliferation and osteoclast differentiation by activating the Wnt/β‐catenin pathway in MM. A) KEGG pathway enrichment analysis for PNPO‐OE versus EV cells was shown as scatter plots. The circle area indicates the number of differentially expressed genes (DEGs) in the pathway, while the circle color represents the range of the corrected *p* values. B) WB assays were performed to detect the levels of MYC, PNPO, and β‐catenin. C) A dual‐luciferase reporter assay revealed that increased PNPO promoted MYC transcription activation (*n* = 3). D) IF analysis showed the translocation of β‐catenin into the nucleus. E) Quantitative analysis data of β‐catenin. F–I) WB assays were used to detect the levels of β‐catenin and PNPO after treatment with MSAB F) or Wnt3a H), and the proliferation capacity was assessed using a CCK8 assay (*n* = 3) G,I). J) GSEA enrichment analysis revealed greater activation of the Wnt/β‐catenin pathway in RAW264.7 PNPO‐OE cells compared to EV cells. K) WB analysis of the levels of β‐catenin and PNPO in RAW264.7 cells. L) A schematic diagram of establishing the adoptive B cell model. M) PNPO overexpression decreased the survival of adoptive B cell model mice (*n* = 12). N) Representative microCT images of bones in the EV and PNPO‐OE groups. O) Representative IHC staining of β‐catenin, PNPO, and MYC in the EV and PNPO‐OE groups. Data are represented as mean ± SD.*p < 0.05, **p < 0.01, ***p < 0.001. Bars represent 2.5 (D) and 100 µm (N).

To elucidate the mechanisms by which PNPO promotes the development of MM cells, we utilized RNA‐sequencing (mRNA‐seq) to identify the activation of PNPO‐related signaling pathways. Our results revealed that the Wnt/β‐catenin pathway is a key pathway (**Figure** **3**A; Figure S2A, Supporting Information). Further WB analysis showed that the levels of β‐catenin and its target gene MYC were significantly altered by PNPO overexpression or knockdown in MM cells (Figure 3B). Moreover, PNPO overexpression increased β‐catenin transcriptional activity (Figure 3C; Figure S2B, Supporting Information) and promoted its nuclear translocation (Figures 3D,E; Figure S2C, Supporting Information). To confirm the involvement of the Wnt/β‐catenin pathway in PNPO‐mediated effects, we used MSAB, a β‐catenin inhibitor, and Wnt3a, a β‐catenin activator, to rescue the effects of PNPO overexpression and knockdown on β‐catenin protein levels and cell proliferation (Figures 3F–I). Interestingly, GSEA enrichment analysis also showed the activation of the Wnt/β‐catenin pathway in RAW264.7 PNPO‐OE cells (Figure 3J; Figure S2D, Supporting Information). Consistent with our findings in MM cells, the protein level of β‐catenin was also correlated with PNPO expression (Figure 3K), and the use of MSAB and Wnt3a reversed the effects of PNPO on β‐catenin protein levels and osteoclasts differentiation (Figures S2E–J, Supporting Information).

MYC^flox/flox^·Cre^+^ mice were injected with NC or PNPO‐OE adenovirus. Spleen cells were screened with B220 beads and subsequently injected into NOD‐SCID mice via the tail vein (Figure 3L). PNPO overexpression decreased the survival of NOD‐SCID mice injected with adoptive B cells (*n* = 12, *p* = 0.0025) (Figure 3M). Tibia µCT analysis showed that bone destruction was significantly more severe in the PNPO‐OE group (*n* = 3, *p* < 0.05) (Figure 3N; Figures S2K, L, Supporting Information). IHC assay showed that PNPO, β‐catenin, and MYC were highly expressed in the PNPO‐OE group, which was also positively correlated with Ki67 and CD138 (Figure 3O; Figure S2M, Supporting Information). In summary, PNPO overexpression activated the Wnt/β‐catenin pathway and exacerbated MM progression.

### 2.4 PNPO Oxidizes DVL3 at the M282 Site, Leading to the Activation of β‐Catenin

**Figure 4:**
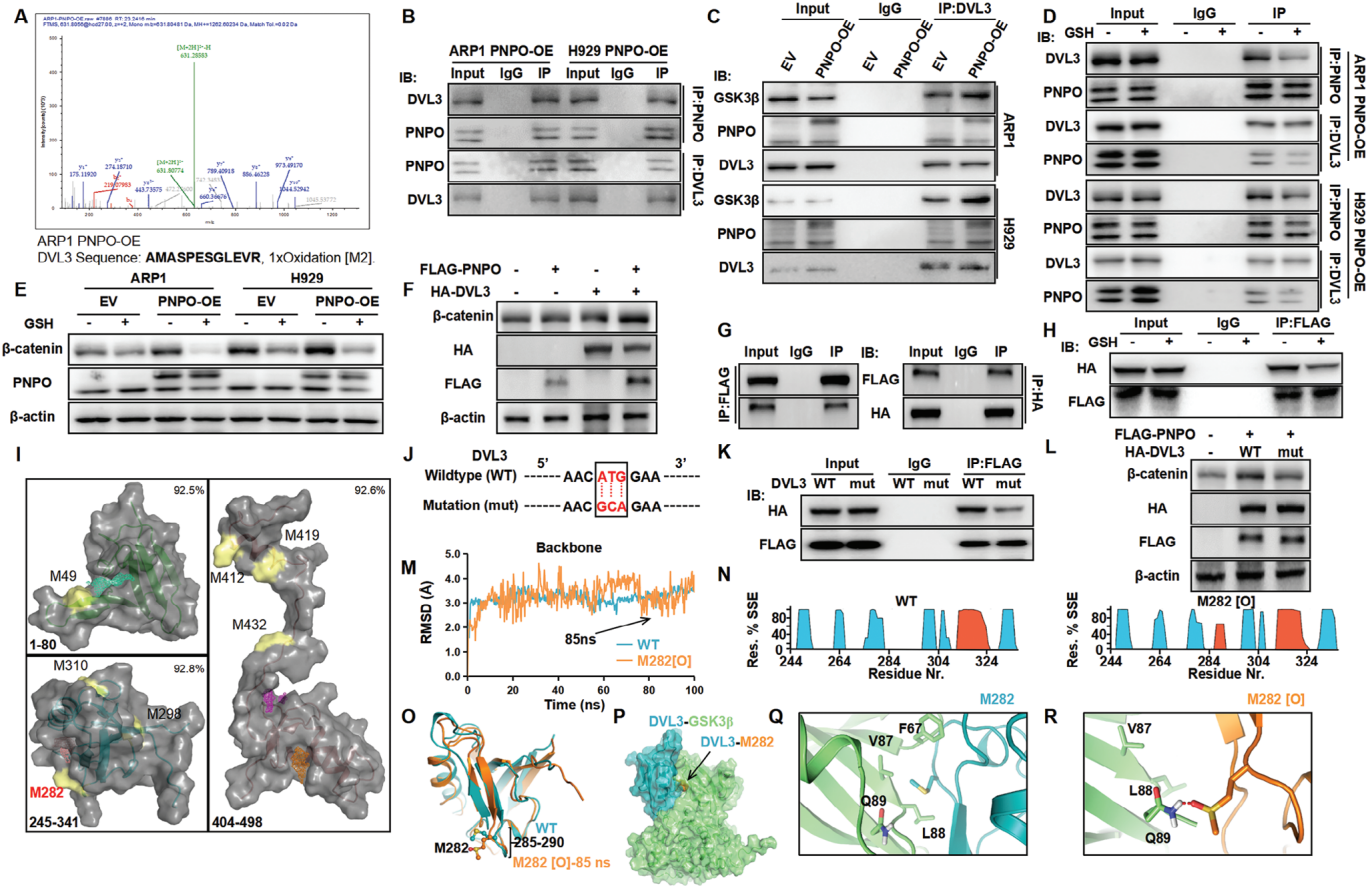
PNPO oxidizes DVL3 at the M282 site, leading to the activation of β‐catenin. A) A secondary MS peak image of DVL3. B) Co‐IP assay confirmed the interaction between DVL3 and PNPO. C) PNPO‐OE cells presented a tighter connection between DVL3 and GSK3β compared to EV cells. D) GSH weakened the binding between PNPO and DVL3. E) WB analysis of β‐catenin and PNPO in MM cells treated with GSH. F) WB analysis showed that β‐catenin expression was increased in HEK‐293 cells co‐transfected with the FLAG‐PNPO and HA‐DVL3 plasmids. G) Co‐IP assay confirmed the interaction between DVL3 and PNPO in HEK‐293 cells. H) GSH weakened the binding between PNPO and DVL3 in HEK‐293 cells. I) Homology models of the DIX domain (left upper), PDZ domain (left lower), and DEP domain (right) of DVL3 were built based on DVL2 crystal structure templates (PDB: 4WIP2, 2REY, and 5SUY4, respectively). The potential binding sites of each domain are represented in green, pink, and orange grids. The superficial methionines are shown on pale‐yellow surfaces. J) The wild‐type sequence of DVL3 and the mutation sequence of DVL3 at methionine 282. K) The DVL3 M282A mutation weakened the interaction between DVL3 and PNPO in HEK‐293 cells. L) WB analysis of β‐catenin with the DVL3 M282A mutation. M) The average root mean square deviation (RMSD) of backbone atoms of the wild‐type DVL3 PDZ domain (WT, blue) and M282 oxidized (M282[O], orange) from molecular dynamics simulations (100 ns). N) SSE‐like alpha‐helices (red) and beta‐strands (cyan) were monitored throughout the simulation. O) Alignment of the wild‐type DVL3 PDZ domain (WT, blue) and M282 oxidized (M282[O], orange) at 85 ns, with residues 285–290 indicated. P) The predicted binding mode of DVL3 (blue) to GSK3β (green), with M282 shown as an orange sphere. Q,R) The detailed binding mode of M282 (blue, Q) and M282[O] (orange, R) interacting with GSK3β (green), with key residues indicated.

Based on the function of PNPO as an oxidase, we hypothesized that it interacts with target proteins and facilitates oxidation. Through MS, we found that PNPO interacted with DVL3, which experienced an oxidation peak in ARP1 PNPO‐OE cells (**Figure** **4**A). To confirm these findings, we conducted a Co‐IP analysis on ARP1 and H929 PNPO‐OE cells, demonstrating the binding between PNPO and DVL3 (Figure 4B). Interestingly, PNPO overexpression increased the connection between DVL3 and GSK3β, resulting in the release of β‐catenin (Figure 4C). Furthermore, we used glutathione (GSH), an antioxidant drug. Pre‐treatment with GSH decreased the binding between PNPO and DVL3 (Figure 4D) and the expression of β‐catenin (Figure 4E), indicating that PNPO interacts with DVL3 and functions as an oxidase.

To further investigate the relationship between PNPO and DVL3, we conducted a co‐transfection experiment in HEK‐293 cells using FLAG‐PNPO and HA‐DVL3, which increased the expression of β‐catenin (Figure 4F). Co‐IP analysis confirmed the interaction between PNPO and DVL3 (Figure 4G). Interestingly, treatment with GSH significantly decreased this interaction (Figure 4H). These findings provide additional evidence of the interaction between PNPO and DVL3 and suggest that its oxidase activity plays a role in this interaction, even in HEK‐293 cells.

At present, there are three functional domains of DVL2: the DIX, PDZ, and DEP domains,^[^ ^29^, ^30^, ^31^ ^]^ which have been predicted to be loop regions by AlphaFold (https://alphafold.ebi.ac.uk/entry/O14641). We built homology models of DVL3 based on DVL2 crystal structure templates, with 92.5%, 92.8%, and 92.6% similarity in the DIX, PDZ, and DEP domains, respectively (Figure S3A, Supporting Information). We identified all the superficial methionines that might be oxidized (Met[O]), resulting in increased binding affinity with GSK3β. Moreover, we also predicted the potential active (binding) sites of each domain (Figure 4I). Interestingly, M282 is located near the active site of the PDZ domain. On this basis, we designed a mutation at M282 to alanine in DVL3 (Figure 4J). As hypothesized, the interaction between PNPO and DVL3 weakened after the M282A mutation (Figure 4K). The expression of β‐catenin was also decreased in HEK‐293 cells co‐transfected with DVL3‐mut (Figure 4L).

We hypothesized that the M282A mutation of DVL3 would decrease its binding with GSK3β. Our initial data showed that the entire molecular dynamics (MD) system remained stable during the simulation, and both DVL3‐WT and M282[O] reached equilibrium after 10 ns of MD runs (Figure 4M). The protein secondary structure elements (Figure 4N) showed an additional alpha‐helices region (285‐290) (Figure 4O) in DVL3 M282[O] compared to WT DVL3. This suggests that differences in residue conformation likely result in altered interaction between DVL3 and GSK3β. Protein‐protein docking simulations reveal that the DVL3 PDZ domain binds to the GSK3β N terminus (Figure 4P), whereas M282 does not interact with GSK3β in a polar manner (Figure 4Q). However, M282[O] forms a hydrogen bond with Q89 (Figure 4R), which may explain the decreased binding affinity with GSK3β. This data indicates that PNPO interacts with and oxidizes DVL3 at M282, a crucial step in DVL3 binding with GSK3β.

### 2.5 Eltrombopag Targets PNPO and Inhibits the Progression of Myeloma

**Figure 5:**
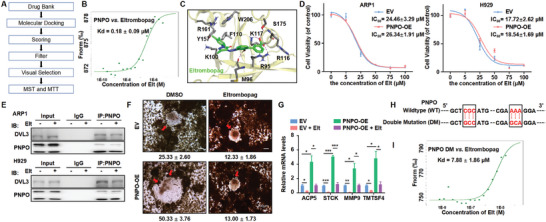
Eltrombopag targets PNPO and inhibits the progression of myeloma. A) The various steps involved in the structure‐based virtual screening workflow. B) MST analysis of the interaction between PNPO protein and Eltrombopag. C) A closer examination of the detailed binding modes of Eltrombopag (green) to PNPO (pale‐yellow cartoon). Key residues are represented as grey sticks, and polar interactions are depicted as magenta dotted lines. D) MTT assay results showed that Eltrombopag inhibited MM cell viability (*n* = 3). E) Eltrombopag weakened the binding between PNPO and DVL3. F) TRAP staining revealed that Eltrombopag inhibited the osteoclast differentiation (*n* = 3). G) The expression of osteoclast‐related mRNAs was detected in RAW264.7 EV and PNPO‐OE cells treated with or without Eltrombopag (*n* = 3). H) Wild‐type and mutated sequences of PNPO at R95 and K115. I) MST analysis of the interaction between PNPO R95A/K117A protein and Eltrombopag. Data are represented as mean ± SD.*p < 0.05, **p < 0.01, ***p < 0.001. Bars represent 200 µm (F).

We established a structure‐based virtual screening workflow (**Figure** **5**A) to identify potential drugs that target the pyridoxal phosphate (PLP)/flavin mononucleotide (FMN) binding site of PNPO. The first step involved the preparation of a focused chemical library, which included only molecules with a maximum of eight rotatable bonds. This focused virtual library was then docked into the PNPO crystal structure via Glide XP docking algorithms. From the docking results, we selected the top 500 poses based on their docking scores and filtered them based on their fundamental interactions, specifically hydrogen bond(s) or ionic bond(s) with R95 and K117. As a result, we identified 14 drug molecules for further analysis after visual inspection.

MST experimental validation revealed that Eltrombopag has a strong affinity for binding to PNPO, with a Kd value of 0.18 ± 0.09 µM (Figure 5B). Further structural analysis (Figures S4A,B, Supporting Information) indicated that Eltrombopag occupies both the PLP and FMN binding sites simultaneously. The dimethyl‐substituted benzene ring of Eltrombopag is located in the PLP site and forms an aromatic interaction with F110 and Y175 of PNPO. The carboxyl group forms an interaction network with R95, K117, and S175, while the phosphate group of FMN also interacts with Eltrombopag. Additionally, the hydroxyl substituent of Eltrombopag interacts with the backbone of M96 and forms a hydrogen bond (Figure 5C).

Eltrombopag inhibited proliferation and induced apoptosis in MM cells (Figure 5D; Figures S4C,D, Supporting Information). In addition, Eltrombopag weakened the binding of PNPO and DVL3 (Figure 5E). Furthermore, Eltrombopag relieved osteoclast differentiation (Figures 5F,G). To further confirm the binding of Eltrombopag with PNPO, we mutated R95/K117 to alanines (Figure 5H) and observed a significant decrease in their interaction (Figure 5I). These results strongly support the potential of Eltrombopag, a PNPO‐targeting drug, as a therapeutic strategy for MM treatment in vitro.

### 2.6 Eltrombopag Inhibits the Proliferation of Myeloma Cells and the Differentiation of Osteoclasts In Vivo

**Figure 6:**
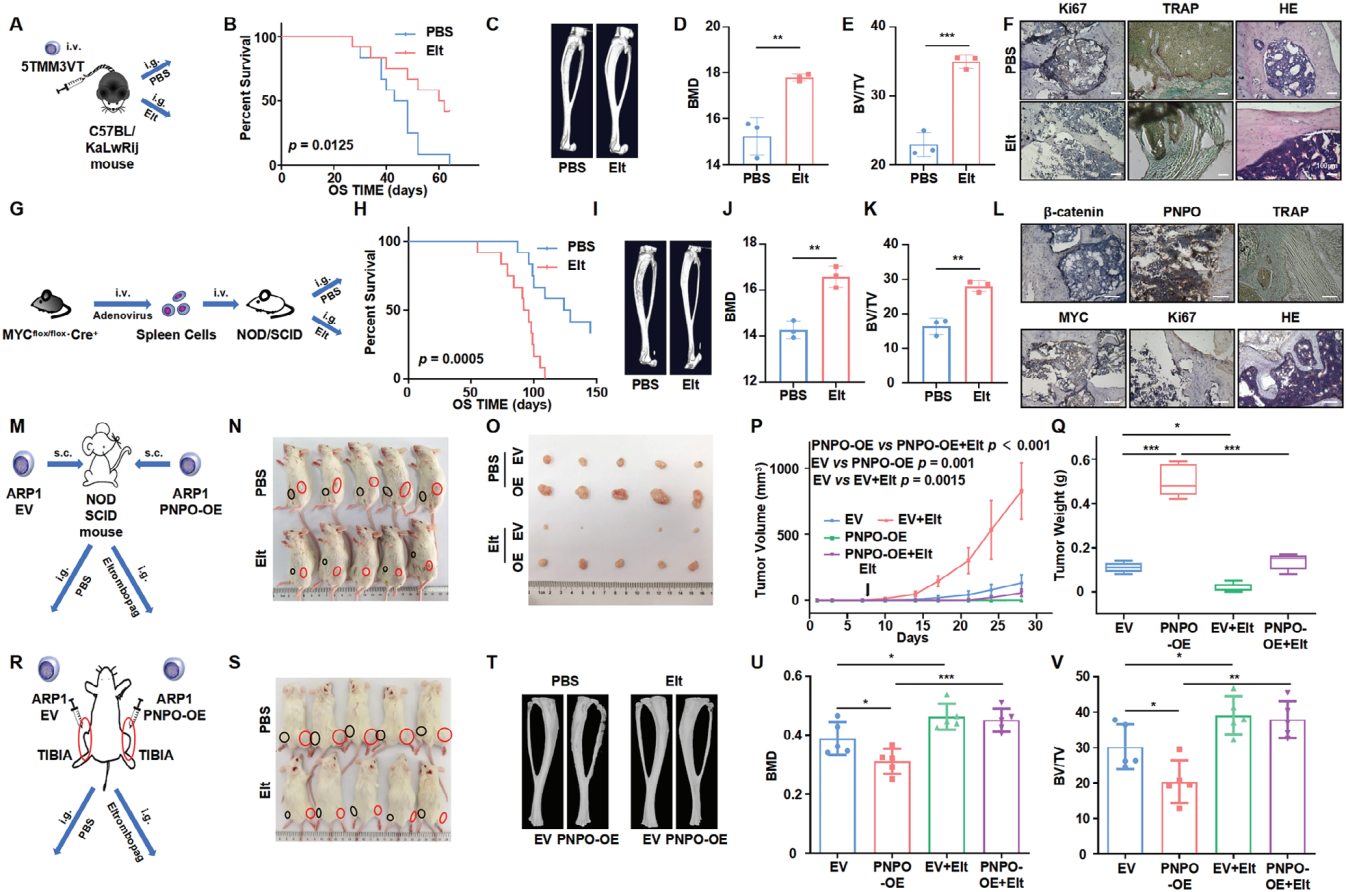
Eltrombopag inhibits the proliferation of myeloma cells and the differentiation of osteoclasts in vivo. A) Schematic diagram of the 5TMM3VT mouse model treated with Eltrombopag. B) Treatment with Eltrombopag significantly prolonged the survival of 5TMM3VT model mice (*n* = 12). C–E) Representative microCT images C), BMD D), and BV/TV E) of 5TMM3VT mouse bones with or without Eltrombopag treatment. F) Representative images of IHC staining for Ki67, HE staining, and TRAP staining in the 5TMM3VT mouse model. G) Schematic diagram of the adoptive B cell model treated with Eltrombopag. H) Eltrombopag treatment significantly prolonged the survival of adoptive B cell model mice (*n* = 12). I–K) Representative microCT images I), BMD J), and BV/TV K) of bones in the adoptive B cell model mice with or without Eltrombopag treatment. L) Representative IHC, HE, and TRAP staining of β‐catenin, PNPO, MYC, and Ki67 in adoptive B cell mouse model. M) Schematic diagram of xenograft mouse model treated with Eltrombopag. N–Q) Photographic images N), schematic images O), the time course of tumor growth P), and tumor weight Q) of xenografts from NOD‐SCID mice with or without Eltrombopag treatment (*n* = 5). R) Schematic diagram of the NOD‐SCID TIBIA mouse model treated with Eltrombopag. S) Photographic images of NOD‐SCID TIBIA mice with or without Eltrombopag treatment (*n* = 5). T–V) Representative microCT images T), BMD U), and BV/TV V) of bones in the NOD‐SCID TIBIA mice with or without Eltrombopag treatment. Data are represented as mean ± SD. *p < 0.05, **p < 0.01, ***p < 0.001. Scale bars represent 100 µm (F,L).

To further investigate the role of Eltrombopag in vivo, we conducted experiments using C57BL/KaLwRij mice injected with 5TMM3VT cells via the tail vein to evaluate its effects (**Figure** **6**A). The results showed that Eltrombopag significantly prolonged the survival (*n* = 12, *p* = 0.0125) and reduced bone damage (*n* = 3, *p* < 0.05) in the 5TMM3VT mouse model (Figures 6B–F). The adoptive B cell model was also used to observe the effect of Eltrombopag on the pathogenesis (Figure 6G). The data revealed that Eltrombopag alleviated the occurrence of MM and prolonged the survival of the model mice (*n* = 12, *p* = 0.0005) (Figure 6H). Tibia µCT analysis showed that Eltrombopag treatment mitigated bone damage (*n* = 3, *p* < 0.05) (Figures 6I–L). We also employed a xenograft model to assess the effect of Eltrombopag on MM cell growth (Figure 6M). After 4 weeks, we visually observed that the tumors derived from ARP1 PNPO‐OE cells grew faster than the tumors derived from ARP1 EV cells, and Eltrombopag inhibited the proliferation of MM cells (*n* = 5, *p* < 0.05) (Figures 6N–Q). Finally, we used the NOD‐SCID TIBIA model to evaluate the effects of Eltrombopag on bone destruction (Figure 6R). The results showed that the upregulation of PNPO promoted osteoclast differentiation, and Eltrombopag significantly reduced bone erosion (*n* = 5, *p* < 0.05) (Figures 6S–V). Our findings suggest that Eltrombopag effectively suppresses MM cell growth and osteoclast differentiation in vivo.

### 2.7 Eltrombopag Effectively Suppresses Tumor Growth in PDX Mouse Model and Patients with MM

**Figure 7:**
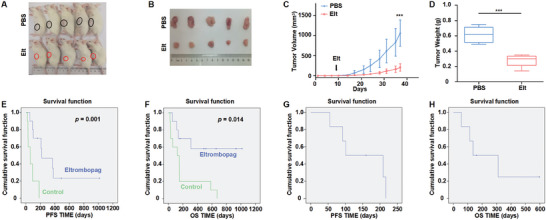
Eltrombopag effectively suppresses tumor growth in PDX mouse models and patients with MM. A–D) The photographic image A), schematic images B), the time course of tumor growth C), and tumor weight D) of PDX NOD‐SCID mice with or without Eltrombopag treatment (*n* = 5). E,F) The mPFS E) and mOS F) rates of patients with or without Eltrombopag treatment (*n* = 10). G,H) The mPFS G) and mOS H) rate of RRMM patients treated with Eltrombopag (*n* = 6). Data are represented as mean ± SD. ***p < 0.001.

To evaluate the effectiveness of Eltrombopag in treating MM at a clinical level, we administered Eltrombopag to PDX mice (**Figure** **7**A). The PDX was implanted into NOD‐SCID mice, and the tumors in the Eltrombopag group were significantly smaller than those in the PBS group (*n* = 5, *p* < 0.001) (Figures 7B–D). Additionally, a series of retrospective clinical trials were conducted to assess the use of Eltrombopag for MM treatment. The baseline characteristics of the patients included in the trials are shown in Table S1 (Supporting Information). Among the ten patients with MM who were treated with Eltrombopag, one achieved complete response (CR), two achieved very good partial response (VGPR), and three achieved partial response (PR), resulting in an objective response rate of 60% compared to the control group, which consisted of patients who did not receive Eltrombopag and had an objective response rate of 10% (*p* = 0.041) (Table S2, Supporting Information). The median progression‐free survival (mPFS) rate was 217.0 days (95% confidence interval (CI): 0–435.625 days) compared to 60.0 days (95% CI: 13.5–106.5 days, *p* = 0.001) (Figure 7E), and the median overall survival (mOS) rate was not reached in patients receiving the combined treatment compared to 122.0 days (95% CI: 0–263.0 days, *p* = 0.014) (Figure 7F). Two of the six patients with recurrent refractory multiple myeloma (RRMM) achieved PR, resulting in an objective response rate of 33.3%. The mPFS rate was 101.0 days (95% CI: 0–213.343 days), and the mOS rate was 136.0 days (95% CI: 0–325.159 days) in patients receiving the combined treatment (Figures 7G,H). These retrospective clinical data suggest that Eltrombopag treatment can potentially alleviate MM progression and improve patient outcomes.

## 3 Discussion

Celastrol, a natural compound extracted from the root extracts of *Tripterygium wilfordii*, is a promising anticancer agent and has attracted the attention of researchers. However, its high toxicity and poor solubility have hindered its use in clinical settings. Intriguingly, we used celastrol as a probe to screen for potential oxidases and identified PNPO as a high‐risk factor in MM. PNPO is involved in protein oxidation, a type of post‐translational modification. Recent research has identified over ten modifications linked to the occurrence and development of cancer, including MM.^[^ ^32^, ^33^, ^34^ ^]^ Oxidation is a well‐known post‐translational modification that is closely associated with redox imbalances in cellular homeostasis.^[^ ^35^ ^]^ These imbalances can significantly impact the fate of cells and their microenvironment. Reactive oxygen species (ROS), which regulate redox reactions, have been found to increase and promote cell proliferation^[^ ^36^ ^]^ and metastasis^[^ ^37^ ^]^ in cancer cells. In addition, ROS can directly oxidize proteins such as GAPDH^[^ ^38^, ^39^, ^40^ ^]^ and pyruvate kinase M2 (PKM2).^[^ ^41^ ^]^ Furthermore, elevated levels of ROS have been shown to stimulate osteoclast differentiation in the BM microenvironment.^[^ ^42^ ^]^ Notably, oxidation is a common post‐translational modification, but other specific sites for oxidizing DVL3 may still need to be explored.

Interestingly, patients with MM have higher ROS levels and protein oxidation compared to healthy individuals and those with MGUS.^[^ ^43^, ^44^, ^45^ ^]^ In our study, we discovered that PNPO oxidizes DVL3 at the M282 site, which increases its binding to GSK3β and prevents β‐catenin from being degraded by the proteasome‐ubiquitin complex. This, in turn, leads to an upregulation of MYC expression. The Wnt/β‐catenin pathway is a well‐conserved pathway whose dysregulation can contribute to the development of cancer.^[^ ^46^ ^]^ However, there are limited studies on the mutations of the Wnt/β‐catenin pathway in MM. The aberrant activation of the Wnt/β‐catenin pathway depends on the decoration of syndecan‐1 by heparan sulfate (HS) proteoglycan,^[^ ^47^, ^48^ ^]^ which upregulates the target genes CCND1 and MYC, serving as essential participants in the pathogenesis of MM.^[^ ^49^, ^50^ ^]^ Additionally, the Wnt/β‐catenin pathway is essential for maintaining bone mass.^[^ ^51^ ^]^ Dickkopf 1 (DKK1) and Wnt5a enhance osteoclast differentiation through the canonical or noncanonical Wnt/β‐catenin pathway.^[^ ^52^, ^53^ ^]^ Our mRNA‐seq data revealed that PNPO overexpression significantly activated the Wnt/β‐catenin signaling pathway, promoting both MM cell proliferation and osteoclast differentiation. Inhibition of β‐catenin^[^ ^54^, ^55^ ^]^ or using small molecule inhibitors, such as ICG‐001^[^ ^56^ ^]^ and BC2059,^[^ ^57^, ^58^ ^]^ has been shown to attenuate MM cell proliferation both in vitro and in vivo. However, the clinical application of these Wnt/β‐catenin inhibitors is limited due to their toxicity and poor bioavailability.^[^ ^59^ ^]^ To address this challenge, we investigated the potential of drug repurposing based on the structure of PNPO, as it plays a role in regulating the Wnt/β‐catenin pathway. Our findings suggest that Eltrombopag may be a promising therapeutic option for MM patients, as it specifically targets the interaction between PNPO and DVL3.

As a nonpeptide thrombopoietin receptor (TpoR) agonist, Eltrombopag has been used in clinical settings to treat primary immunologic thrombocytopenic purpura (ITP)^[^ ^60^, ^61^ ^]^ and severe aplastic anemia (AA).^[^ ^62^ ^]^ In MM, thrombocytopenia is a common complication that leads to poor prognosis.^[^ ^63^ ^]^ Treatment with TPO can suppress MM progression,^[^ ^63^ ^]^ suggesting that Eltrombopag may be a potential drug for MM. Interestingly, Eltrombopag has also been found to inhibit TET dioxygenase^[^ ^64^ ^]^ and rapidly decrease ROS levels in various cells,^[^ ^65^ ^]^ indicating its potential to improve oxidative stress through ROS and related oxidases. Our data showed that Eltrombopag disrupted the binding between PNPO and DVL3 by directly binding to PNPO. Through molecular docking simulations via computer technology, we found that Eltrombopag tightly binds to the R95 and K117 sites of PNPO. We also conducted in vivo and in vitro experiments to demonstrate that Eltrombopag could intervene in MM cell proliferation and bone damage. While Eltrombopag has been used in MM patients with thrombocytopenia, its impact on patient survival has yet to be thoroughly researched. Our study found that Eltrombopag had positive effects on treating MM in both a PDX mouse model and in clinical patients. However, further verification is needed to confirm these findings due to the limited sample size. It is also important to consider the potential risk of thrombosis associated with the use of Eltrombopag, as it has been shown to have off‐target effects such as iron chelation.^[^ ^66^ ^]^ Therefore, larger and more comprehensive clinical trials are necessary to fully evaluate the efficacy and safety of Eltrombopag for MM, particularly with respect to its potential for thrombosis. In future studies, we will focus on reconstructing and optimizing the structure of Eltrombopag to increase its therapeutic effectiveness. This may involve modifying specific chemical groups or adjusting the dosage to minimize potential off‐target effects.

In conclusion, our data indicate that PNPO is a significant risk factor for the development of MM. Our findings suggest that PNPO promotes cell proliferation in MM and osteoclast differentiation through Exos. Mechanistically, PNPO increases the level of β‐catenin by oxidizing DVL3, which then recruits GSK3β. Furthermore, our research demonstrates that Eltrombopag has the potential to inhibit the proliferation of myeloma cells and osteoclast differentiation, indicating that it may be a promising treatment for MM in the future.

## 4 Experimental Section

### Antibodies and Reagents

Antibodies were used as follows: PNPO (E‐8) (sc‐393561 Santa Cruz Biotechnology); DYKDDDDK (also called FLAG) (14793S, Cell Signaling Technology); cyclin B1 (4135S, Cell Signaling Technology); poly ADP‐ribose polymerase (PARP) (9542S, Cell Signaling Technology); caspase 3 (9665, Cell Signaling Technology); cleaved caspase 3 (9661L, Cell Signaling Technology); Alix (2171S, Cell Signaling Technology); CD9 (13174S, Cell Signaling Technology); β‐actin (66009‐1‐Ig, ProteinTech Group); β‐catenin (51067‐2‐AP, ProteinTech Group); DVL3 (13444‐1‐AP, ProteinTech Group); HA Tag (51064‐2‐AP, ProteinTech Group); GSK3β (22104‐1‐AP, ProteinTech Group); Histone H3 (17168‐1‐AP, ProteinTech Group); PNPO (15552‐1‐AP, ProteinTech Group); MYC (10828‐1‐AP, ProteinTech Group); calnexin (10427‐2‐AP ProteinTech Group); Rabbit IgG (a7016, Beyotime); Mouse IgG (a7028, Beyotime); Goat anti‐Rabbit IgG(H+L) HRP (FMS‐Rb01, Fcmacs); Goat anti‐Mouse IgG (H+L) HRP (S0002, Affinity); Goat pab to Ms IgG (FITC) (ab6785, Abcam); Goat Anti‐Mouse IgG (H+L) Fluor647‐conjugated (S0013, Affinity); Ki67 (af1098, Affinity). Puromycin was obtained from Merck KGaA. Doxycycline (Dox) (ST039A) was purchased from Beyotime. GSH (s0073) was purchased from Longwave. Wnt3a (HZ‐1296) was sourced from Proteintech. Diphenyltetrazolium Bromide (MTT) (M8180) was obtained from Solarbio. Cell counting Kit‐8 (CCK8) (K1018) was purchased from APExBIO. Methyl 3‐{[(4‐methylphenyl) sulfonyl] amino} benzoate (MSAB) (S6901) was purchased from Selleck. Eltrombopag (GC12513) was obtained from Good Laboratory Practice Biosciences.

### Cell Lines and Culture

Two human myeloma cell lines (HMCLs), ARP1 and H929, were selected for studies on overexpression (OE) and inducible knockdown (KD) of PNPO. All cell lines were cultured under common in vitro conditions of 37 °C and 5% CO_2_. ARP1 and H929 cells were cultured in RPMI‐1640 (Biological Industries), while HEK‐293 and RAW264.7 cells were cultured in DMEM (Biological Industries). Each culture medium was supplemented with 10% fetal bovine serum (Gibco), 100 U mL^−1^ penicillin, and 100 µg mL^−1^ streptomycin (Sigma Aldrich).

### Plasmids, Transfection, and Small Interfering RNA

A plasmid containing a fragment of the human PNPO cDNA sequence was inserted into the CD513B‐1 vector with GFP green fluorescence and 3 × FLAG tags (Quanyang Biological Co.). The fragment containing human PNPO‐targeting shRNA, with a Dox‐inducible promoter, was cloned and inserted into the pTRIPZ vector. The PNPO‐OE or PNPO‐KD plasmid was co‐transfected with the packaging plasmids PLP1, PLP2, and VSVG (in a ratio of 3:1:1:1) into HEK‐293 cells by using Hieff TransTM Liposomal Transfection Reagent (40802ES03, YEASEN). After 48 h, the lentivirus supernatant was harvested. The transfected MM cells were then screened with puromycin. A plasmid containing a fragment of the mouse PNPO cDNA sequence was inserted into the pcDNA3.1 vector with FLAG tags (Fenghui). Mouse PNPO‐targeting small interfering (si) RNA (GGGAGUACCUGAGAAAGAATT) was obtained from GenePharma. The efficiency of transfection and transduction was determined by WB assay.

### High‐Throughput Sequencing

ARP1, H929, RAW264.7 empty vector (EV), and PNPO‐OE cells were stored in TRIeasy (19201ES60, YEASEN). The subsequent mRNA‐seq experiments were conducted by Lianchuan Biotechnology Co., Ltd. with the relevant data available through the NCBI GEO database (GSE230387 and GSE193820). After conducting SDS‐PAGE gel electrophoresis, the entire gel was stained with Coomassie Bright Blue for 1 h and then allowed to elute overnight. The electrophoresis strips were then removed and sent to Lianchuan Biotechnology Co., Ltd. for mass spectrum (MS) analysis. The relevant data were accessible through the ProteomeXchange Consortium (http://proteomecentral.proteomexchange.org) via the iProX partner repository (PXD027698).

### Animal Models

The pristane‐induced model was utilized to examine the effect of PNPO on spontaneous plasma cell tumors. Adeno‐associated virus, labeled with green fluorescent protein and HA, was intravenously injected into 4–5‐week‐old Balb/c mice (both female and male) through the tail vein. At the same time, pristane was administered three times at a 2‐month interval via intraperitoneal injection. Spleens were collected for protein expression analysis, ascites cells were collected to detect CD138 expression, and serum samples were used to check for abnormalities.

The adoptive B cell model was utilized to investigate the impact of PNPO and MYC on the development of MM. The adeno‐associated virus was injected into MYC^flox/flox^·Cre^+^ mice (5–6 weeks old, female or male) through the tail vein, and their spleen cells were then isolated using CD45R (B220) MicroBeads (130‐049‐501, Miltenyi Biotec). After three weeks, these cells were injected into NOD‐SCID mice through the tail vein. The survival time was recorded, and the bone density and volume of the tibias were analyzed using micro‐computed tomography (µCT, SkyScan1176, Bruker microCT).

A xenograft model was utilized to assess the growth of MM cells in vivo. H929 PNPO‐KD cells (1.5 × 10^6^) were subcutaneously injected into NOD‐SCID mice (5–6 weeks old, male), and Dox (2 mg mL^−1^) was administered through the drinking water, starting on day 5 after the transfer of myeloma cells. Similarly, to evaluate the impact of Eltrombopag on MM cell growth, 1.5 × 10^6^ ARP1 EV and PNPO‐OE cells were injected into the left and right flanks of NOD‐SCID mice (5–6 weeks old, male), respectively. Eltrombopag (2 mg kg^−1^) was administered twice a week through intragastric injection. The mice were euthanized with pentobarbital through intraperitoneal injection when the tumors reached a size of 15 mm. Tumor tissues were collected, weighed, and photographed. The tumor volume was calculated via the following formula: tumor volume = 0.5 × Length × Width × Width.

The NOD‐SCID TIBIA model was utilized to evaluate bone damage. A total of 1 × 10^7^ ARP1 EV or PNPO‐OE cells were injected into the BM cavity of the tibias in NOD‐SCID mice (5–6 weeks old, male). To assess osteolysis, the bone density and volume of the tibias were measured via µCT.

The 5TMM3VT mouse model was utilized to assess the efficacy of Eltrombopag in promoting survival. Specifically, 1 × 10^6^ 5TMM3VT cells were injected intravenously into the tail vein of C57BL/KaLwrij mice (4–5 weeks old, male). The mice were then administered Eltrombopag (2 mg kg^−1^) twice a week until they were either sacrificed or deceased. The survival time was recorded, and osteolysis was evaluated via µCT.

The efficacy of Eltrombopag was evaluated in a patient‐derived tumor xenograft (PDX) model. Biopsy samples were obtained from an extramedullary tumor under the skin of an MM patient at the Department of Hematology, First Affiliated Hospital of Nanjing Medical University. These samples were then transplanted into NOD‐SCID mice (5–6 weeks old, male) subcutaneously and harvested once they reached a size of 500 mm^3^. The tumor tissues were then divided into 2.5 × 2.5 × 2.5 mm^3^ pieces and implanted subcutaneously again. This process was repeated three times. The mice were then randomly assigned to receive treatment with or without Eltrombopag (2 mg kg^−1^), once the tumor size reached 100–150 mm^3^.

All the animal studies were conducted according to the Government‐published recommendations for the Care and Use of Laboratory Animals and received approval from the Institutional Ethics Review Boards of Nanjing University of Chinese Medicine (Ethics Registration no. 202204A020).

### Human Subjects

A study on the use of Eltrombopag in patients with MM was conducted by the Blood Alliance of Yangtze‐River‐Delta Country, which includes Shanghai Jing'an District Zhabei Central Hospital, Zhangjiagang First People's Hospital, Qingpu Branch of Zhongshan Hospital, Chongming Hospital Affiliated to Shanghai University of Medicine & Health Sciences, Yancheng No.1 People's Hospital, as well as Shanghai Fourth People's Hospital. The study was conducted in accordance with the Declaration of Helsinki 2013 and was approved by the hospital's institutional review board. Informed consent was obtained from all patients (Ethics Registration no. ZBLL2023071306001).

### GEP Analysis

The gene expression profiling (GEP) cohorts were collected from the NCBI GEO database as previously described.^[^ ^67^, ^68^ ^]^ The included datasets were Total Therapy 2 (TT2, GSE2658), the MGUS dataset (GSE5900), the newly diagnosed MM patient dataset (GSE136337), the assessment of proteasome inhibition for extending remission (APEX) patient cohort (GSE9728), and the bone lesions described dataset (GSE6401). The patients were divided into different groups based on disease progression or the presence of bone lesions, and the differences in PNPO expression were further examined using ANOVA or *t*‐test. To generate the Kaplan–Meier survival curve of patients, X‐tile software was utilized, and statistical significance was determined using the Log‐rank test.

### Cell Viability, Cell Cycle, and Apoptosis Assays

Trypan blue staining was used to count cells using a hemocytometer to assess cell viability. The cells were then seeded at a density of 1 × 10^3^ per well in 96‐well plates. After 24, 48, and 72 h, the OD450 absorbance with CCK8 or OD570 absorbance with MTT was measured using Varioskan LUX 6–1536 (Thermo Scientific, Rockford). For the cell cycle assay, cells were resuspended in 70% ethanol at −20 °C overnight. Ribonuclease A (10405ES03, YEASEN) was added 1 h before resuspending the cells. The cells were then stained with propidium iodide (PI) (c0080, Solarbio LIFE SCIENCES) for 15 min at room temperature. Flow cytometry was performed using the Guava easyCyteTM system from Merck Millipore in Germany. For cell apoptosis assay, cells were treated with either Dox or Eltrombopag. Apoptosis was measured by labeling cells with APC‐conjugated AnnexinV (B317159; Biolegend) in combination with PI. The resulting cells were analyzed using GuavaSoft, and the data were processed through FlowJo.

### Quantitative PCR

Total RNA was extracted from RAW264.7 cells by using TRIzon Reagen (CW0580S, CWBIO). Complementary DNA was synthesized using HiFiScript gDNA Removal RT MasterMix (CW2020 M, CWBIO). Quantitative PCR (qPCR) was performed using SuperStar Universal SYBR Master Mix (CW3360 M, CWBIO) with GAPDH as the loading control. The qPCR was conducted using Analytikjena qPCR soft 4.0 with the following program: pre‐denaturation at 95 °C for 3 min, denaturation at 95 °C for 10 s, annealing at 60 °C for 60 s, and a total of 40 cycles. The relative expression levels of the target genes were calculated using the 2^−ΔΔCT^ method and graphed as fold change relative to the control. The sequences of primers can be found in Table S3 (Supporting Information).

### IF Staining and Confocal Microscopy

Cells were fixed with 4% paraformaldehyde, permeabilized with PBS containing 0.1% Triton X‐100, and blocked with 1% BSA. After overnight incubation with primary antibodies at 4 °C, slides were incubated with corresponding secondary antibodies. Images were captured using a confocal microscope (TCS SP8, Leica).

### Nuclear and Cytoplasmic Protein Extraction

Nuclear and cytoplasmic protein extraction was conducted using a Nuclear and Cytoplasmic Protein Extraction Kit (P0027, Beyotime) following the manufacturer's instructions.

### Dual‐Luciferase Reporter Assay

To conduct the dual‐luciferase reporter assay, the relevant plasmids were co‐transfected into HEK‐293 cells and incubated for 24 h. The assay was performed via a Dual Glo Luciferase Reporter Gene Assay Kit (11405ES, YEASEN) according to the manufacturer's instructions.

### Co‐Immunoprecipitations and Western Blotting

Co‐immunoprecipitations (Co‐IPs) using the Pierce Direct Magnetic IP/Co‐IP Kit (88828, Thermo Scientific) and antibodies against PNPO, DVL3, FLAG, and HA were used as recently described. IgG antibodies were used as a negative control. Whole MM cell lysates were prepared using RIPA lysis buffer (20101ES60, YEASEN). To separate proteins (10 µg per sample), 8–15% SDS‐PAGE gels were used and blocked with 5% non‐fat milk in Tris‐buffered saline containing 0.5% Tween‐20. The proteins were transferred to polyvinylidene fluoride membranes and incubated with a primary antibody (1:1000) overnight at 4 °C. Afterward, they were incubated with peroxidase‐conjugated horseradish peroxidase for 1 h at 4 °C and displayed via an automatic gel imaging analysis system (JC‐1075p, Peiqing Science and Technology Co., Ltd.).

### Exosome Isolation and Confirmation

The supernatant from ARP1 EV or PNPO‐OE cells was collected and centrifuged using the following steps: 300 × g for 10 min, 2000 × g for 10 min, and 10 000 × g for 30 min to remove floating cells and debris. The remaining supernatant was centrifuged in an ultracentrifuge at 100 000×g for 70 min. The collected precipitate was washed with PBS and centrifuged at 100 000×g for 70 min. The precipitate was resuspended in 200 µL of PBS and stored at −80 °C. The morphology was examined via a JEM‐2100 transmission electron microscope (JEOL). The markers Alix and CD9 and the negative marker calnexin were detected using WB assay.

### Tartrate‐Resistant Acid Phosphatase Activity Staining

RAW264.7 cells were seeded in a 24‐well plate at a density of 3000 cells per well. The culture medium was supplemented with recombinant murine sRANKL (50 ng mL^−1^) and M‐CSF (10 ng mL^−1^) and changed every other day. After 6 days, the Leukocyte Tartrate‐resistant acid phosphatase (TRAP) kit (387A, Sigma Aldrich) was used to stain the cells and detect TRAP activity. Finally, the samples were stained with 1% aqueous Fast Green FCF for 1 min. The numerical values at the bottom of the figures indicate the number of differentiated osteoclasts in one well of a 24‐well plate for three repeated experiments, which was recorded as the red multinucleate fusion count.

### Mutation

All mutation experiments were conducted via the Mut Express II Fast Mutagenesis Kit V2 (C214, Vazyme) following the instructions provided by the manufacturer. The sequences of the mutation primers can be found in Table S4 (Supporting Information).

### Microscale Thermophoresis

The PNPO WT and double‐mutation (DM) proteins were purified using the pet‐28a vector plasmid with a 6×His tag through HisTrap HP (17‐5248‐02, GE Healthcare). The proteins were then labeled with Monolith RED‐NHS (M0‐L011, Nano Temper, Germany) and analyzed via microscale thermophoresis (MST) (NT.115, Nano Temper).

### Construction and Refinement of the DVL3 Structure

All the crystal structures were retrieved from the Protein Data Bank (https://www.rcsb.org/) and were prepared via molecular operating environment (MOE) software (Chemical Computing Group, Inc.: Montreal, Canada). The sequence of DVL3 was obtained from Uniprot^[^ ^69^ ^]^ and aligned with the crystal structure sequence of DVL2. 3D models of DVL3 were then constructed via MOE software, based on the crystal structures prepared for DVL2. Molecular dynamics simulations were performed via the Desmond module^[^ ^70^ ^]^ from Schrödinger to refine these models. Equilibrium MD simulation was implemented for 100 ns, resulting in a trajectory with 500 unique conformations. Default settings were used for all other parameters. The analysis module in Desmond was used to generate the trajectory.

### Prediction of DVL3 and GSK3β Binding Modes

The binding site of the DVL3 structure, consisting of the DIX, PDZ, and DEP domains, was predicted via the Schrödinger SiteMap algorithm (version 4.6.011).^[^ ^71^, ^72^ ^]^ Protein‐protein docking was performed via Schrödinger to predict the binding modes of DVL3 and GSK3β. DVL3 was designated as the ligand and GSK3β as the receptor, with M282 being constrained as “attractive.”

### Virtual Screening in Drug Discovery to Identify Potential Drug Candidates

Compounds were downloaded from the Drugbank database^[^ ^73^ ^]^ (https://go.drugbank.com/) and saved in SMILES format. The OPLS3 force field^[^ ^74^ ^]^ was used to prepare the database by performing energy minimization and generating possible protonation states for each compound at pH 7.0 ± 2.0 via the LigPrep tool of Schrödinger 2018‐1 (version 45011, Schrödinger, LLC, https://www.schrodinger.com/). The crystal structure of PNPO (PDB: 1NGR11) was obtained from the Protein Data Bank. Several necessary steps were performed to prepare the proteins for further study, including splitting chains, removing water and ions, and performing 3D protonation. After eliminating unnecessary components such as chains, ligands, ions, and water, the remaining crystal structures were preprocessed via the “Protein Preparation Wizard” module of Schrödinger 2018‐1, with all the parameters set to default values. The co‐crystallized ligand was used to define the pockets as the center point by utilizing the “Receptor Grid Generation” function. All the molecular docking simulations were conducted via Glide (version 78011; Schrödinger, Inc., NY, 2013). The prepared compounds and grid files generated up to 30 poses for each ligand in Glide XP mode. The docking score for each ligand was calculated by adding the Glide G score and the state penalty for a specific protonation or tautomeric state. In addition to the comprehensive docking score, Glide utilized the Emodel scoring function to assess the validity of different docking poses for protein‐ligand complexes. After applying a key interaction‐based filter based on forming hydrogen bonds/ionic interactions with R95/K117 and visually inspecting the results, 14 drugs were selected for further experimental validation.

### Immunohistochemical Staining

Immunostaining was performed on 3‐µm paraffin‐embedded tissue sections mounted on 3‐aminopropyltriethoxysilane‐coated slides. The slides were incubated with primary antibodies at 4 °C overnight, and the secondary antibody was incubated for 45 min at 37 °C, followed by the addition of the streptavidin‐biotin complex at 37 °C for 30 min, the addition of 3,3′‐diaminobenzidine for color development, and finally counterstaining with hematoxylin. An experimental pathologist performed the PNPO and Ki67 staining semiquantitative measurements. The score depended on the following staining intensity scores: 0 (negative), 1+ (weak), 2+ (moderate), and 3+ (strong), multiplied by the cell number (0–100%) at each intensity level. The final staining score was subsequently calculated by summing the four intensity percentages (0–300).

### Statistical Analysis

All the statistical results were expressed as mean ± SD. The statistical analysis was performed via IBM SPSS Statistics 19 or GraphPad Prism 8 software. Differences between the two groups were compared via a two‐tailed Student's *t*‐test. One‐way analysis of variance (ANOVA) with Tukey's post hoc comparison was used for comparisons between more than two groups. The survival of MM patients was determined via Kaplan–Meier curve and Log‐rank test. In all analyses, *p*‐values were two‐tailed, and values less than 0.05 were considered statistically significant. The following symbols were used to indicate the level of significance: *p for *p* < 0.05, **p for *p* < 0.01, ***p for *p* < 0.001, and n.s. for no significance.

## Conflict of Interest

The authors declare no conflict of interest.

## Author Contributions

Z.D., S.S., and N.Z. contributed equally to this work. C.G., Y.Y., N.L., and F.Z. designed and supervised the projects. Z.D. and S.S. drafted the manuscript and created the figures. C.G. and Y.Y. revised the manuscript and figures. Z.D., S.S., Y.P., L.C., X.Y., and Y.X.Y. performed the experiments and analyzed the data. M.G. provided technical support. N.Z., M.X., Y.C., and F.Z. provided support for the collection and analysis of clinical samples from MM patients. All the authors have read and approved the final version of the manuscript. All the listed authors have read and approved the final version, including all details and images.

## Supporting information



Supporting Information

## Data Availability

The published article includes all data generated or analyzed during this study and a summary of the data in the accompanying tables, figures, and supplemental materials. The RNA‐seq data (GSE230387 and GSE193820) and MS data (PXD027698) have been deposited at GEO or ProteomeXchange Consortium and are publicly available as of the publication date.
