# Supplementary material for: PNPO‐Mediated Oxidation of DVL3 Promotes Multiple Myeloma Malignancy and Osteoclastogenesis by Activating the Wnt/β‐Catenin Pathway
Source: Adv Sci (Weinh). 2024 Dec 10;12(5):2407681. doi: 10.1002/advs.202407681 (PMC11792023; doi:10.1002/advs.202407681)
Supplement: Supplementary file 1 — Supporting Information [file ADVS-12-2407681-s001.docx]

**Supplemental Information**

**PNPO-mediated Oxidation of DVL3 Promotes Multiple Myeloma Malignancy and Osteoclastogenesis by Activating the Wnt/β-catenin Pathway**

*Zhendong Deng,^#^ Shanliang Sun,^#^* *Nian Zhou,^#^* *Yumeng Peng, Long Cheng, Xichao Yu, Yuxia Yuan, Mengjie Guo, Min Xu, Yuexin Cheng,* *Fan Zhou,^*^ Nianguang Li,^*^ Ye Yang,^*^ and* *Chunyan Gu^*^*

**Table S1 The baseline characteristics of MM patients with Eltrombopag treatment.**

| Characteristic | Eltrombopag  Value (n = 10) | Control  (untreated with Eltrombopag)  Value (n = 10) | *p* Value |
| --- | --- | --- | --- |
| Age |  |  |  |
| Median(range)-year | 65.5(52-72) | 64(49-80) |  |
| Distribution-no. (%) |  |  | 0.656 |
| < 65yr | 4(40) | 6(60) |  |
| ≥65yr | 6(60) | 4(40) |  |
| Male sex-no. (%) | 5(50) | 6(60) | 1.000 |
| ECOG performance-status score-no. (%) |  |  | 0.033 |
| 0-2 | 5(50) | 0(0) |  |
| 3-4 | 5(50) | 10(100) |  |
| R-ISS disease stage |  |  | 1.000 |
| I | 0(0) | 0(0) |  |
| II | 6(60) | 5(50) |  |
| III | 4(40) | 5(50) |  |
| Chromosomal abnormality-no. (%) |  |  |  |
| High risk overall | 6(60) | 6(60) | 1.000 |
| del(17p)/p53 | 2(20) | 2(20) | 1.000 |
| t (4;14) | 2(20) | 1(10) | 0.176 |
| t (14;16) | 0(0) | 0(0) |  |
| gain(1q) | 4(40) | 5(50) | 1.000 |
| Extramedullary infiltration-no. (%) | 7(70) | 2(20) | 0.070 |
| Median time since initial diagnosis (range) - month | 57(0.5-146) | 41.5(1-141) | 0.384 |
| Median no. of previous treatment regimens (range) | 3(1-6) | 2.5(1-4) | 0.969 |
| Median no. of previous recurrence (range) | 2(0-6) | 2.5(1-4) | 0.316 |

**Table S2 Clinical benefits of MM patients with Eltrombopag treatment.**

| Treatment efficacy | Eltrombopag  Value (n = 10) | Control  (untreated with Eltrombopag)  Value (n = 10) | *p* Value |
| --- | --- | --- | --- |
| ≥PR | 6(60) | 1(10) | 0.041 |
| sCR | 0 | 0 |  |
| CR | 1(10) | 0 |  |
| VGPR | 2(20) | 1(10) |  |
| PR | 3(30) | 0 |  |
| MR | 0 | 0 |  |
| SD | 3(30) | 5(50) |  |
| PD | 1(10) | 4(40) |  |

**Table S3 The sequences of the primers used in the study.**

|  | | Sequence (5’-3’) |
| --- | --- | --- |
| ACP5 | Forward primer | *GCGACCATTGTTAGCCACATACG* |
|  | Reverse primer | *CGTTGATGTCGCACAGAGGGAT* |
| STCK | Forward primer | *AGCAGAACGGAGGCATTGACTC* |
|  | Reverse primer | *CCCTCTGCATTTAGCTGCCTTTG* |
| MMP9 | Forward primer | *GCTGACTACGATAAGGACGGCA* |
|  | Reverse primer | *TAGTGGTGCAGGCAGAGTAGGA* |
| TMTSF4 | Forward primer | *TTTGCCGCTGTGGACTATCTGC* |
|  | Reverse primer | *GCAGAATCATGGACGACTCCTTG* |
| GAPDH | Forward primer | *CATCACTGCCACCCAGAAGACTG* |
|  | Reverse primer | *ATGCCAGTGAGCTTCCCGTTCAG* |

**Table S4 The specific mutation primers designed for this study.**

|  | | Sequence (5’-3’) |
| --- | --- | --- |
| DVL3 M282A | Forward primer | *CACTCTCAACGCAGAAAAATATAACTTCTTGGGCATCTCC* |
|  | Reverse primer | *TTTCTGCGTTGAGAGTGACCGTGATGATGTTG* |
| PNPO R95A | Forward primer | *AAAACCCTCTGCTgcCATGTTGCTGCTGAAGGGCT* |
|  | Reverse primer | *ATGgcAGCAGAGGGTTTTCCATCTCTG* |
| PNPO K117A | Forward primer | *AGAGTCGAgcAGGAAAAGAGCTGGACTCTAATCCC* |
|  | Reverse primer | *CTTTTCCTgcTCGACTCTCGAAGTTAGTGAAGAAGC* |


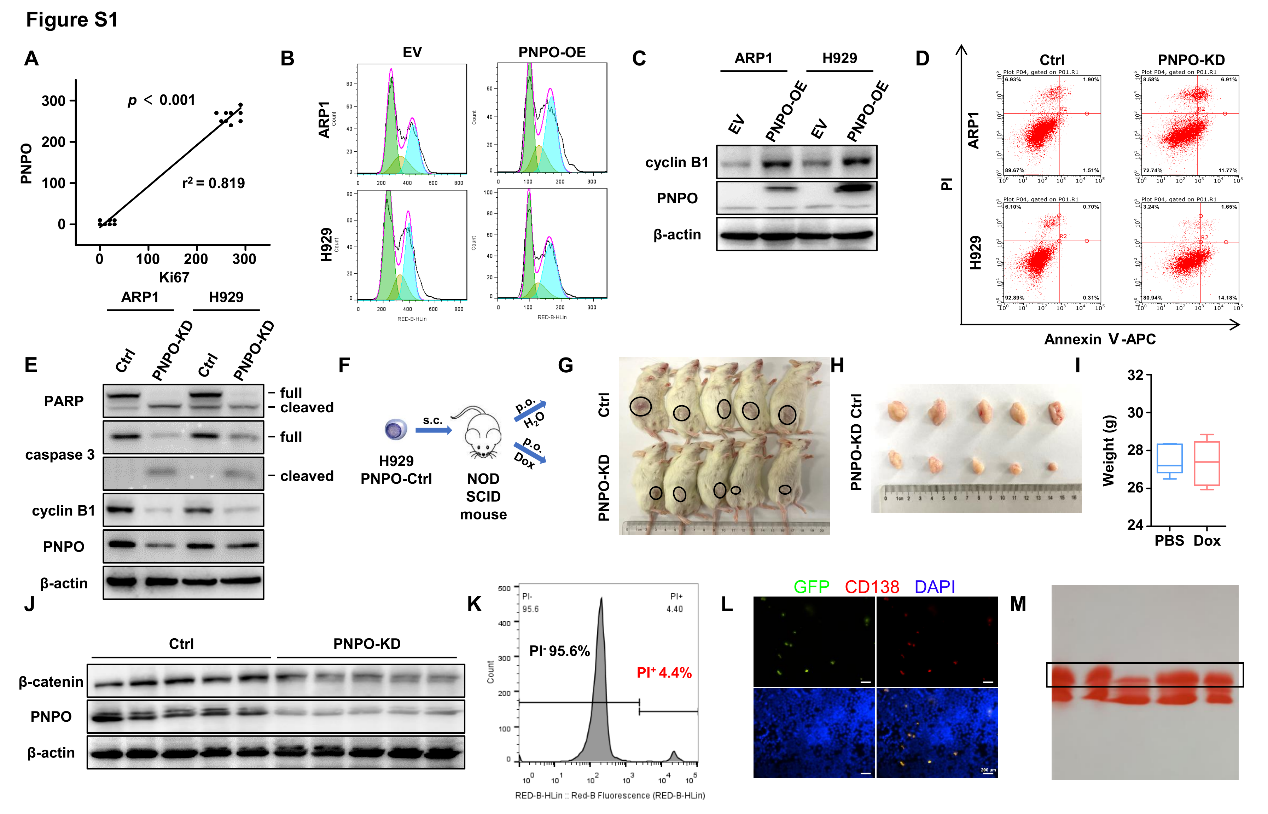


**Figure S1 PNPO is a high-risk indicator for poor prognosis and promotes cell proliferation in MM.** A) IHC assay demonstrated a positive correlation between PNPO and Ki67, a key marker of cell proliferation. B) Cell cycle analysis was performed on EV and PNPO-OE cells (n = 3). C) WB analysis showed increased levels of cyclin B1 in MM PNPO-OE cells compared to EV cells. D) Cell apoptosis analysis was conducted on Ctrl and PNPO-KD cells (n = 3). E) WB analysis revealed decreased levels of cyclin B1 and increased levels of cleaved-caspase3 and cleaved-PARP in MM PNPO-KD cells compared to Ctrl cells. F) A schematic diagram of establishing the xenograft model (n = 5). G-I) Photographic images G), schematic images H), and weight I) of the xenograft mice were captured at the end of the study. J) WB analysis showed increased levels of PNPO and β-catenin in tumor tissues from the xenograft model. K) Flow cytometry analysis showed that most ascites cells were live cells. L) IF analysis revealed CD138 expression in ascites cells. M) Serum protein electrophoresis (SPEP) showed abnormal levels in most mice. Data are represented as mean ± SD. **p* < 0.05, ***p* < 0.01, ****p* < 0.001. The scale bar represents 200 μm (L).


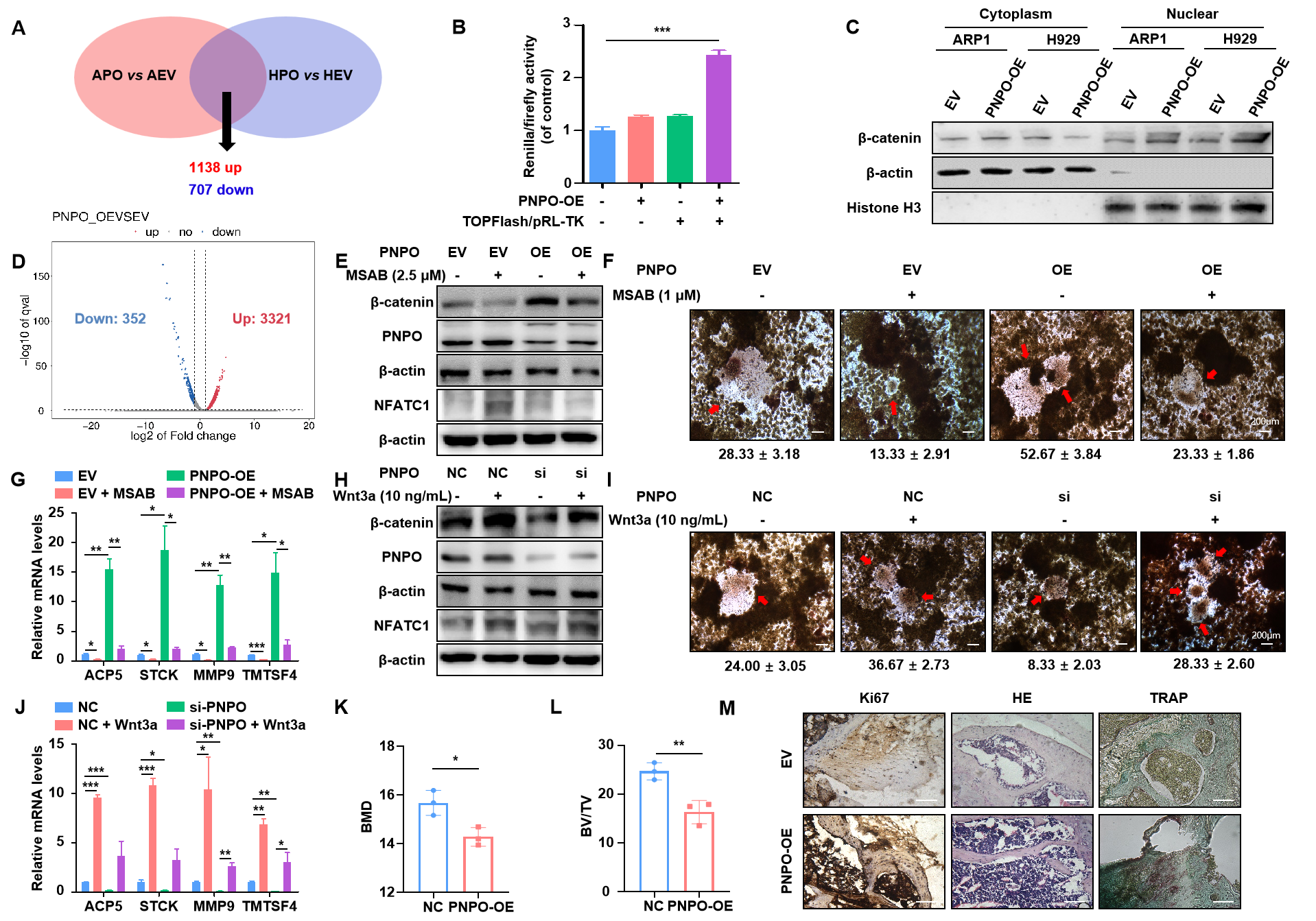


**Figure S2 PNPO affects cell proliferation and osteoclast differentiation by activating the Wnt/β-catenin pathway in MM.** A) A Venn diagram revealed 1138 up-regulated genes and 707 down-regulated genes in PNPO-OE cells compared to EV MM cells. B) A dual-luciferase reporter assay revealed that overexpressed PNPO enhanced the transcription ability of β-catenin (n = 3). C) Analysis of nuclear and cytoplasmic protein extraction showed that β-catenin translocated into the nucleus. D) Scatter plot diagram revealed 3321 up-regulated genes and 352 down-regulated genes in PNPO-OE RAW264.7 cells compared to EV cells. E) WB analysis of β-catenin, PNPO, and NFATC1 with treatment of MSAB in PNPO-OE RAW264.7 cells. F) TRAP staining showed that MSAB relieved the differentiation of RAW264.7 cells into osteoclasts (n = 3). G) The expression of osteoclast-related mRNAs was detected in RAW264.7 EV and PNPO-OE cells treated with or without MSAB (n = 3). H) WB analysis of β-catenin, PNPO, and NFATC1 with the treatment of Wnt3a in si-PNPO RAW264.7 cells. I) TRAP staining results revealed that Wnt3a rescued the differentiation of RAW264.7 cells into osteoclasts (n = 3). J) The expression of osteoclast-related mRNAs was detected in RAW264.7 si-NC and si-PNPO cells treated with or without Wnt3a (n = 3). K) Bone mineral density (BMD) and L) Bone volume/total volume (BV/TV) of adoptive B cell model mice (n = 5). M) Representative IHC staining of Ki67, HE staining, and TRAP staining in EV and PNPO-OE groups. Data are represented as mean ± SD.**p* < 0.05, ***p* < 0.01, ****p* < 0.001. Bars represent 200 μm (F and I) and 100 μm (M).


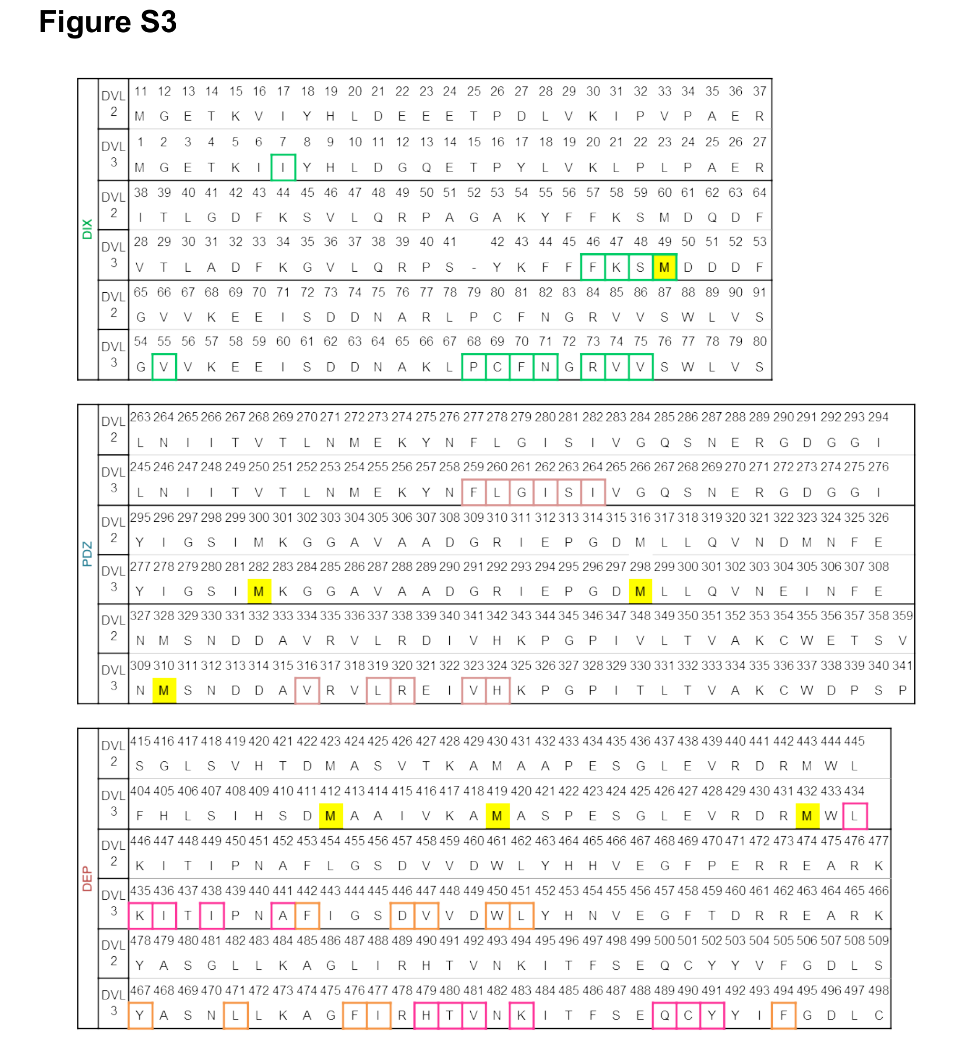


**Figure S3 Sequence alignment of the DIX, PDZ, and DEP domains of DVL2 and DVL3.** Residues buried within their corresponding sites are indicated by color-coded boxes, while related methionines are highlighted with an orange background.


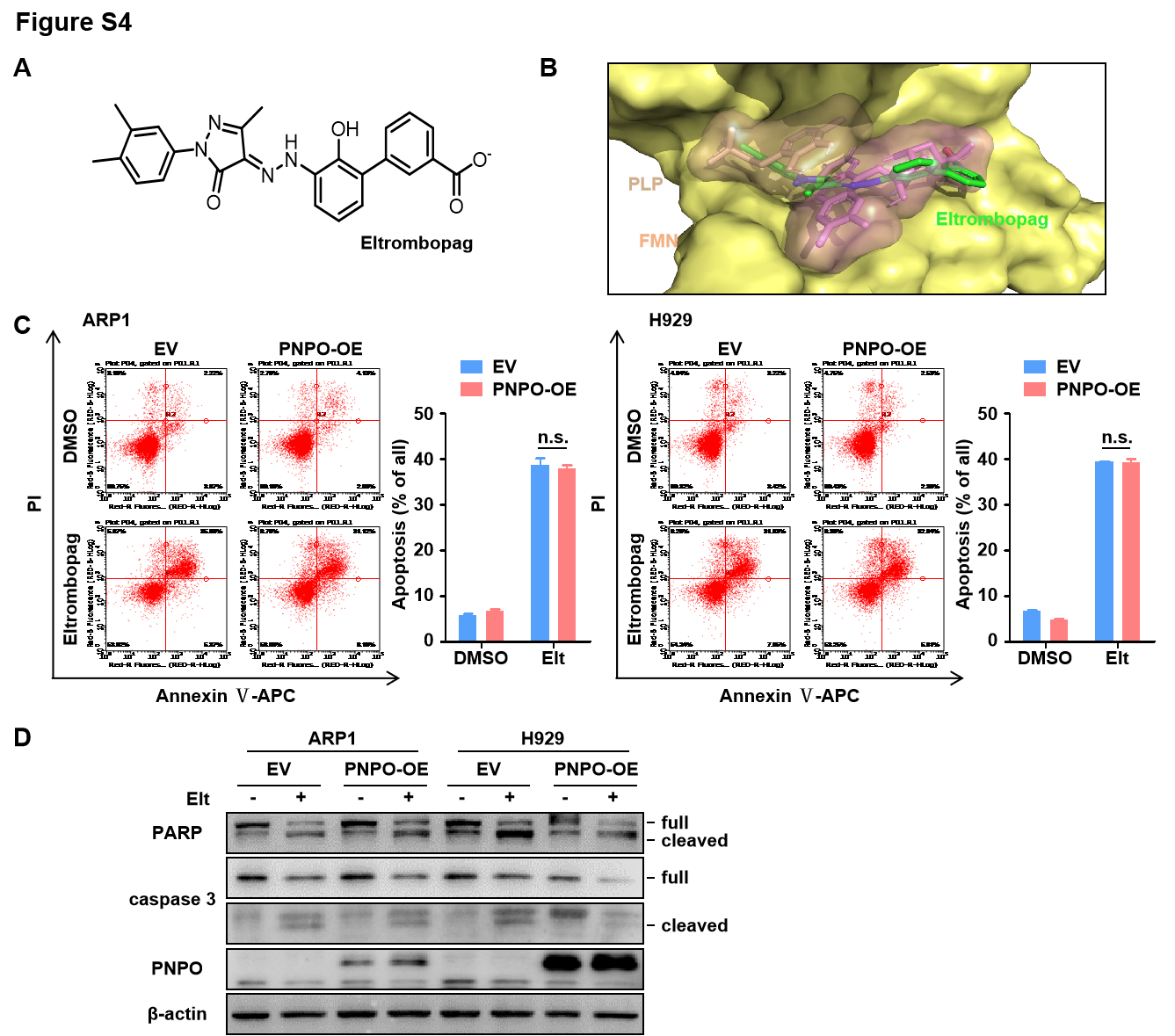


**Figure S4 Eltrombopag targets PNPO and inhibits the progression of myeloma.** A) The chemical structure of Eltrombopag. B) The compound Eltrombopag (depicted as green sticks) is aligned with the co-crystallized ligand PLP (represented in wheat) as well as FMN (illustrated by ping). PNPO, PLP, and FMN are also shown as corresponding transparent surfaces. C) Cell apoptosis analysis for MM cells treated with Eltrombopag (n = 3). D) WB analysis of caspase3 and PARP in MM cells treated with Eltrombopag. Data are represented as mean ± SD. **p* < 0.05, ***p* < 0.01, ****p* < 0.001.
